# Supplementary material for: Genetic and clinical landscape of breast cancers with germline BRCA1/2 variants
Source: Commun Biol. 2020 Oct 16;3:578. doi: 10.1038/s42003-020-01301-9 (PMC7567851; doi:10.1038/s42003-020-01301-9)

Supplementary Information for

Genetic and clinical landscape of breast cancers with germline *BRCA1/2* variants

Yukiko Inagaki-Kawata[1,2], Kenichi Yoshida[1], Nobuko Kawaguchi-Sakita[3], Masahiro Kawashima[2], Tomomi Nishimura[1,2], Noriko Senda[2], Yusuke Shiozawa[1], Yasuhide Takeuchi[1, 15, 16], Yoshikage Inoue[1], Aiko Sato-Otsubo[1], Yoichi Fujii[1], Yasuhito Nannya[1], Eiji Suzuki[2], Masahiro Takada[2], Hiroko Tanaka[18], Yuichi Shiraishi[4], Kenichi Chiba[4], Yuki Kataoka[5], Masae Torii[6], Hiroshi Yoshibayashi[6], Kazuhiko Yamagami[7], Ryuji Okamura[8], Yoshio Moriguchi[9], Hironori Kato[10], Shigeru Tsuyuki[11], Akira Yamauchi[12], Hirofumi Suwa[13], Takashi Inamoto[14], Satoru Miyano[4,18], Seishi Ogawa[1, 15, 17] and Masakazu Toi[2]

1. Department of Pathology and Tumor Biology, Kyoto University, Kyoto, Japan; 2. Department of Breast Surgery, Kyoto University, Kyoto, Japan; 3. Department of Clinical Oncology, Kyoto University Hospital, Kyoto, Japan; 4. Laboratory of DNA Information Analysis, Human Genome Center, Institute of Medical Science, The University of Tokyo, Tokyo, Japan; 5. Hospital Care Research Unit/ Department of Respiratory Medicine, Hyogo Prefectural Amagasaki General Medical Center, Amagasaki, Japan; 6. Department of Breast Surgery, Japanese Red Cross Wakayama Medical Center, Wakayama, Japan; 7. Department of Breast Surgery, Shinko Hospital, Kobe, Japan; 8. Department of Breast Surgery, Yamatotakada Municipal Hospital, Yamatotakada, Japan; 9. Department of Breast Surgery, Kyoto City Hospital, Kyoto, Japan; 10. Department of Breast Surgery, Kobe City Medical Center General Hospital, Kobe, Japan; 11. Department of Breast Surgery, Osaka Red Cross Hospital, Osaka, Japan; 12. Department of Breast Surgery, Kitano Hospital, Osaka, Japan; 13. Department of Breast Surgery, Hyogo Prefectural Amagasaki General Medical Center, Amagasaki, Japan; 14. Tenri Health Care University, Tenri, Japan; 15. Institute for the Advanced Study of Human Biology (WPI-ASHBi), Kyoto University, Kyoto, Japan; 16. Department of Diagnostic Pathology, Kyoto University, Kyoto, Japan; 17. Department of Medicine, Centre for Haematology and Regenerative Medicine, Karolinska Institute, Stockholm, Sweden; 18 Laboratory of Sequence Analysis, Human Genome Centre, Institute of Medical Science, The University of Tokyo, Tokyo, Japan

Correspondence to:
Prof. Seishi Ogawa
Pathology and Tumor Biology, Kyoto University, Yoshida-Konoe-cho, Sakyo-ku, Kyoto, 606-8501, Japan.
Phone: +81-75-753-9285; E-mail: sogawa-tky@umin.ac.jp

Prof. Masakazu Toi
Breast Cancer Unit, Kyoto University Hospital
Breast Surgery, Graduate School of Medicine, Kyoto University
54 Shogoin-Kawaracho, Sakyo-ku, Kyoto 606-8507, Japan
Phone: +81-75-751-3660; E-mail: toi@kuhp.kyoto-u.ac.jp

Supplementary Table

Supplementary Table 1. Pathogenic germline variants identified in 1,995 breast cancer patients.

Supplementary Table 2. Characteristics of patients carrying germline variants for each gene.

Supplementary Table 3. Germline mutations in subgroups according to family history of breast cancer and ovarian cancer.

Supplementary Table 4. Multivariate analysis of survival in breast cancer patients.

Supplementary Table 5. Genes for targeted sequencing of tumor samples.

Supplementary Table 6. Driver mutations identified in 60 breast tumors.

Supplementary Table 7. The landscape of driver mutations in patients who have germline *BRCA1/2* variants.

Supplementary Table 8. Clinical correlates with *BRCA1/2* biallelic inactivation.

Supplementary Table 9. Multivariate analysis of survival with biallelic inactivation of *BRCA1/2* as a covariate.

Supplementary Figure legends:

Supplementary Figure 1. Study design.

a. A total of 2,136 breast cancer patients were enrolled in this study who were treated through Kyoto Breast Cancer Research Network institutions. Among these, 1,995 cases fulfilled the inclusion criteria. **b.** Flow chart of judgement of pathogenic germline variants.

Supplementary Figure 2. Pathogenic mutations identified in 11 breast cancer susceptibility genes in 1,995 unselected breast cancer patients.

Distribution of pathogenic variants on each gene. Locations of identified pathogenic variants are shown with the protein domains shown by lollipop structures. Variant types are indicated by colors.

Supplementary Figure 3. Clinical characteristics of breast tumors harboring pathogenic germline variants.

a. Distribution of age at diagnosis in patients according to the mutated genes (below). **b.** Frequency of germline mutations by the age at onset in 15-year-age groupings. **c.** Distribution of T factor by 3 age groupings and the status of germline mutation. **d.** Distribution of N factor according to the status of germline mutation.

Supplementary Figure 4. Disease-free and overall survival of patients with and without germline variants.

a. Kaplan-Meier disease-free survival curve for patients with and without germline variants. **b.** Kaplan-Meier overall survival curve for patients with and without germline variants.

Supplementary Figure 5. Genetic lesions associated with germline *BRCA1/2* mutations and biallelic inactivation.

a. Mutational signatures identified in breast tumors with *BRCA1/2* germline mutations: signature characterized by T>C mutations caused by microsatellite instability (Sig_1), age-related C>T mutations at CpG sites (Sig_2), mutational signature caused by deficient HR (Sig_3), and APOBEC-related signature (Sig_4). **b.** Number of single nucleotide variants (SNVs) and indels, and proportion of each mutational signature in tumors with *BRCA1/2* germline mutations. **c.** Relationship between the number of Sig_3 mutations and status of *BRCA1/2* mutations and biallelic inactivation in TCGA dataset (n = 778), including *BRCA*(-) (n = 744), *BRCA2* tumor with (n = 14) and without (n = 4) biallelic inactivation and *BRCA1* tumor with (n = 15) and without (n = 1) biallelic inactivation. **d.** Number and types of SVs in in tumors with *BRCA1/2* germline mutations. **e.** Relationship between the number of deletions and tandem duplications and status

of *BRCA1/2* mutations and biallelic inactivation in TCGA dataset (n = 778). **f.** Frequently mutated driver genes in breast tumors with *BRCA1/2* germline mutations with and without biallelic inactivation.

Supplementary Figure 6. VAFs of *TP53* and *RB1* mutations with *BRCA1/2*-mutated tumors with LOH.

a. VAFs of mutations on chromosome 17 of tumors with germline *BRCA1* mutations and LOH in TCGA cohort. VAFs of *TP53* mutations are shown in red dotted lines. **b.** VAFs of mutations on chromosome 13 of tumors with germline *BRCA2* mutations and LOH in TCGA cohort. VAFs of *RB1* mutations are shown in red dotted lines.

Supplementary Figure 7. Disease-free and overall survival of *BRCA1/2*-mutated patients with and without biallelic inactivation.

a. Kaplan-Meier disease-free survival curve for *BRCA1/2*-mutated patients with/without biallelic inactivation and those without germline mutations in our data set. **b.** Kaplan-Meier overall survival curve for *BRCA1/2*-mutated patients with/without biallelic inactivation and those without germline mutations in our data set.

Supplementary Figure 8. Comparison with the previous study.

For TCGA samples, our study (Inagaki-Kawata et al.,) and the previous study (Maxwell et al.) analyzed 24 samples in common, for which results of both studies are compared. Thirteen samples were exclusively analyzed by Maxwell et al., for which exclusion criteria in our study are shown.

Supplementary Figure 9. Validation of mutation and copy number calling

a. Comparison of mutations called by EBCall and MuTect. Distribution of VAFs of mutations called by both methods and those called by either one of the two are shown in right. **b.** Identification of gain with LOH based on the estimated tumor purities by total and allele specific (As) copy number (CN). **c.** Correlation of the LOH status of *BRCA1/2* loci determined by CNACS and Control-FREEC.

Supplementary Figure 10. Comparison of copy number analysis by CNACS and SNP array

Total and allele specific copy numbers of 5 tumors estimated by sequencing (CNACS) and SNP array karyotyping are shown. Regions with LOH determined by SNP array are shown in orange bars.

Supplementary Table 1. Pathogenic germline variants identified in 1,995 breast cancer patients

No. of patients	Chr	Start	End	Ref	Alt	Gene	Function	AAChange
1	11	108160488	108160488	C	T	*ATM*	Nonsense SNV	NM_000051:exon29:c.C4396T:p.R1466X
1	11	108202644	108202647	TTTG	-	*ATM*	Frameshift deletion	NM_000051:exon52:c.7668_7671del:p.T2556fs
1	11	108202673	108202676	CAAA	-	*ATM*	Frameshift deletion	NM_000051:exon52:c.7697_7700del:p.A2566fs
1	17	41197784	41197784	G	A	*BRCA1*	Nonsense SNV	NM_007294:exon23:c.C5503T:p.R1835X
1	17	41215382	41215382	G	A	*BRCA1*	Nonsense SNV	NM_007294:exon18:c.C5161T:p.Q1721X
1	17	41222967	41222967	G	A	*BRCA1*	Missense SNV	NM_007294:exon15:c.C4964T:p.S1655F
1	17	41243025	41243026	CT	-	*BRCA1*	Frameshift deletion	NM_007294:exon11:c.4120_4121del:p.S1374fs
1	17	41243777	41243778	CT	-	*BRCA1*	Frameshift deletion	NM_007294:exon10:c.3770_3771del:p.E1257fs
1	17	41244135	41244135	C	-	*BRCA1*	Frameshift deletion	NM_007294:exon10:c.3413delG:p.G1138fs
1	17	41244426	41244426	G	C	*BRCA1*	Nonsense SNV	NM_007294:exon10:c.C3122G:p.S1041X
2	17	41244748	41244748	G	A	*BRCA1*	Nonsense SNV	NM_007294:exon10:c.C2800T:p.Q934X
1	17	41245279	41245279	C	-	*BRCA1*	Frameshift deletion	NM_007294:exon10:c.2269delG:p.V757fs
2	17	41258497	41258497	A	T	*BRCA1*	Nonsense SNV	NM_007294:exon4:c.T188A:p.L63X
2	17	41267797	41267797	C	T	*BRCA1*	Splice site	NM_007294:exon3:c.81-1G>A
1	17	41276047	41276047	-	T	*BRCA1*	Frameshift insertion	NM_007294:exon2:c.66dupA:p.E23fs
1	13	32893406	32893407	CT	-	*BRCA2*	Frameshift deletion	NM_000059:exon3:c.260_261del:p.T87fs
1	13	32893464	32893464	T	C	*BRCA2*	Splice site	NM_000059:exon3:c.316+2T>C
1	13	32899298	32899298	-	CTAAAT	*BRCA2*	Frameshift insertion	NM_000059:exon4:c.402_403insCTAAATTC:p.L134fs
1	13	32903578	32903578	A	G	*BRCA2*	Splice site	NM_000059:exon8:c.632-2A>G
1	13	32906628	32906628	C	-	*BRCA2*	Frameshift deletion	NM_000059:exon10:c.1013delC:p.A338fs
1	13	32906889	32906889	A	-	*BRCA2*	Frameshift deletion	NM_000059:exon10:c.1274delA:p.E425fs
1	13	32907014	32907014	A	T	*BRCA2*	Nonsense SNV	NM_000059:exon10:c.A1399T:p.K467X
3	13	32907421	32907421	A	-	*BRCA2*	Frameshift deletion	NM_000059:exon10:c.1806delA:p.G602fs
1	13	32911133	32911133	G	T	*BRCA2*	Nonsense SNV	NM_000059:exon11:c.G2641T:p.E881X
3	13	32911298	32911301	AAAC	-	*BRCA2*	Frameshift deletion	NM_000059:exon11:c.2806_2809del:p.K936fs
1	13	32911684	32911687	AATT	-	*BRCA2*	Frameshift deletion	NM_000059:exon11:c.3192_3195del:p.S1064fs
2	13	32911725	32911725	T	-	*BRCA2*	Frameshift deletion	NM_000059:exon11:c.3233delT:p.V1078fs
1	13	32912432	32912432	A	T	*BRCA2*	Nonsense SNV	NM_000059:exon11:c.A3940T:p.K1314X
1	13	32913030	32913033	ATGA	-	*BRCA2*	Frameshift deletion	NM_000059:exon11:c.4538_4541del:p.D1513fs
1	13	32913464	32913464	C	T	*BRCA2*	Nonsense SNV	NM_000059:exon11:c.C4972T:p.Q1658X
1	13	32913971	32913975	ATTAA	-	*BRCA2*	Frameshift deletion	NM_000059:exon11:c.5479_5483del:p.I1827fs
13	13	32914066	32914069	AATT	-	*BRCA2*	Frameshift deletion	NM_000059:exon11:c.5574_5577del:p.T1858fs
2	13	32914110	32914113	TAAT	-	*BRCA2*	Frameshift deletion	NM_000059:exon11:c.5618_5621del:p.V1873fs
1	13	32914137	32914137	C	A	*BRCA2*	Nonsense SNV	NM_000059:exon11:c.C5645A:p.S1882X
2	13	32914459	32914460	AG	-	*BRCA2*	Frameshift deletion	NM_000059:exon11:c.5967_5968del:p.S1989fs
3	13	32914894	32914898	TAACT	-	*BRCA2*	Frameshift deletion	NM_000059:exon11:c.6402_6406del:p.N2134fs
1	13	32914974	32914977	ACAA	-	*BRCA2*	Frameshift deletion	NM_000059:exon11:c.6482_6485del:p.D2161fs
1	13	32915042	32915042	C	T	*BRCA2*	Nonsense SNV	NM_000059:exon11:c.C6550T:p.Q2184X
1	13	32915089	32915089	T	-	*BRCA2*	Frameshift deletion	NM_000059:exon11:c.6597delT:p.T2199fs
1	13	32915109	32915112	AAAC	-	*BRCA2*	Frameshift deletion	NM_000059:exon11:c.6617_6620del:p.K2206fs
1	13	32915141	32915141	A	T	*BRCA2*	Nonsense SNV	NM_000059:exon11:c.A6649T:p.K2217X
5	13	32920978	32920978	C	T	*BRCA2*	Nonsense SNV	NM_000059:exon13:c.C6952T:p.R2318X
1	13	32929289	32929308	AAAGCA	-	*BRCA2*	Frameshift deletion	NM_000059:exon14:c.7299_7318del:p.Q2433fs
1	13	32930609	32930609	C	T	*BRCA2*	Nonsense SNV	NM_000059:exon15:c.C7480T:p.R2494X
1	13	32930687	32930687	C	T	*BRCA2*	Nonsense SNV	NM_000059:exon15:c.C7558T:p.R2520X
1	13	32931938	32931939	TT	-	*BRCA2*	Frameshift deletion	NM_000059:exon16:c.7677_7678del:p.S2559fs
1	13	32937362	32937362	A	G	*BRCA2*	Missense SNV	NM_000059:exon18:c.A8023G:p.I2675V
1	13	32945095	32945095	G	A	*BRCA2*	Nonsense SNV	NM_000059:exon20:c.G8490A:p.W2830X
1	13	32945141	32945141	G	T	*BRCA2*	Nonsense SNV	NM_000059:exon20:c.G8536T:p.E2846X
1	13	32950810	32950811	AC	-	*BRCA2*	Frameshift deletion	NM_000059:exon21:c.8636_8637del:p.N2879fs
1	13	32954009	32954009	C	T	*BRCA2*	Nonsense SNV	NM_000059:exon23:c.C9076T:p.Q3026X
1	13	32954050	32954050	G	A	*BRCA2*	Synonymous SNV	NM_000059:exon23:c.G9117A:p.P3039P
1	22	29085179	29085179	G	A	*CHEK2*	Nonsense SNV	NM_007194:exon14:c.C1486T:p.Q496X
1	22	29121089	29121089	G	C	*CHEK2*	Nonsense SNV	NM_007194:exon4:c.C468G:p.Y156X
1	22	29130478	29130478	G	A	*CHEK2*	Nonsense SNV	NM_007194:exon2:c.C232T:p.Q78X
1	17	29528171	29528171	-	T	*NF1*	Frameshift insertion	NM_000267:exon10:c.1180dupT:p.H393fs
1	16	23619279	23619279	G	A	*PALB2*	Nonsense SNV	NM_024675:exon12:c.C3256T:p.R1086X
1	16	23619288	23619289	CA	-	*PALB2*	Frameshift deletion	NM_024675:exon12:c.3246_3247del:p.S1082fs
1	16	23632748	23632749	AA	-	*PALB2*	Frameshift deletion	NM_024675:exon10:c.3047_3048del:p.F1016fs
1	16	23646314	23646314	G	C	*PALB2*	Nonsense SNV	NM_024675:exon4:c.C1553G:p.S518X
1	16	23646315	23646316	AT	-	*PALB2*	Frameshift deletion	NM_024675:exon4:c.1551_1552del:p.K517fs
2	16	23646636	23646636	T	A	*PALB2*	Nonsense SNV	NM_024675:exon4:c.A1231T:p.R411X
1	16	23646810	23646811	TC	-	*PALB2*	Frameshift deletion	NM_024675:exon4:c.1056_1057del:p.E352fs
1	16	23652472	23652472	C	A	*PALB2*	Nonsense SNV	NM_024675:exon1:c.G7T:p.E3X
1	10	89623773	89623774	GA	-	*PTEN*	Frameshift deletion	NM_001304717:exon1:c.67_68del:p.E23fs
1	10	89685300	89685300	C	A	*PTEN*	Nonsense SNV	NM_001304717:exon4:c.C714A:p.Y238X
1	10	89692905	89692905	G	A	*PTEN*	Missense SNV	NM_001304717:exon6:c.G908A:p.R303Q
1	10	89720852	89720852	C	T	*PTEN*	Nonsense SNV	NM_001304717:exon9:c.C1522T:p.R508X
1	17	7574017	7574017	C	T	*TP53*	Missense SNV	NM_000546:exon10:c.G1010A:p.R337H
1	17	7578211	7578211	C	T	*TP53*	Missense SNV	NM_000546:exon6:c.G638A:p.R213Q
1	17	7578388	7578388	C	T	*TP53*	Missense SNV	NM_000546:exon5:c.G542A:p.R181H
1	17	7578457	7578457	C	T	*TP53*	Missense SNV	NM_000546:exon5:c.G473A:p.R158H

Supplementary Table 2. Characteristics of patients carrying germline variants for each gene

		Germline mutation(+) (N=101)								Germline mutation (-) (N=1,894)	All (N=1,995)
		BRCA2 (N=62)	*BRCA1* (N=15)	*PALB2* (N=9)	*CHEK2* (N=3)	*TP53* (N=4)	*PTEN* (N=4)	*ATM* (N=3)	*NF1* (N=1)		
Age	～35	8	5	0	1	1	0	0	0	36	51
	36～45	16	3	2	0	0	2	0	1	293	317
	46～55	6	2	3	1	1	0	1	0	447	461
	56～65	20	3	1	1	0	1	0	0	496	522
	66～	12	2	3	0	2	1	2	0	622	644
Histology	IDC	48	11	8	2	1	4	1	1	1408	1484
	ILC	6	1	0	1	0	0	0	0	66	74
	DCIS	6	1	1	0	3	0	1	0	200	212
	others	2	2	0	0	0	0	1	0	101	106
Phenotype	ER +	50	4	8	3	2	3	3	1	1466	1540
	ER -	10	11	1	0	1	1	0	0	353	377
	HER2 +	6	1	1	0	1	0	1	0	300	310
	HER2 -	51	13	6	3	1	4	2	1	1301	1382
	Ki67 high (>14)	40	13	6	1	2	0	1	1	903	967
	Ki67 low	10	0	0	2	0	4	1	0	704	721
Histological grade	3	9	9	4	1	0	0	1	1	121	146
	2	19	2	1	2	1	1	0	0	393	419
	1	9	0	1	0	0	0	1	0	206	217
Clinical stage	0	6	1	1	0	3	1	1	0	241	254
	I	20	2	2	0	0	3	0	0	747	774
	II	26	9	2	3	1	0	0	1	691	733
	III	5	2	1	0	0	0	2	0	132	142
	IV	4	0	1	0	0	0	0	0	48	53
	Unknown	1	1	2	0	0	0	0	0	35	39
PH	Ovarian cancer	3	1	0	0	0	0	0	0	10	14
FH	Breast cancer	28	5	1	2	1	1	1	0	309	348
	Ovarian cancer	3	0	0	0	0	0	0	0	30	33

Abbreviations: IDC, Invasive ductal carcinoma ; ILC, Invasive lobular carcinoma ; DCIS, Ductal carcinoma in situ ; ER, Estrogen receptor ; HER2, Human epidermal growth factor receptor 2 ; PH, Past histroy ; FH, Family history

Supplementary Table 3. Germline mutations in subgroups according to family history of breast cancer and ovarian cancer

		Mutation cases			
	Patients, N	*BRCA1* (%)	*BRCA2* (%)	Others (%)	Total (%)
FH+	372	5 (1.3)	30 (8.0)	6 (1.6)	41 (11.0)
FH-	1,455	7 (0.5)	31 (2.1)	12 (0.8)	50 (3.4)
NA	168	3	1	6	10

Abbreviations: FH, Family history

Supplementary Table 4. Multivariate analysis of survival in breast cancer patients

	OS			DFS		
	Hazard ratio	P-value	95% CI	Hazard ratio	P-value	95% CI
Germline mutation positive	0.44	0.21	0.071-1.5	0.64	0.21	0.28-1.3
larger than T3	3.3	0.0020	1.6-6.8	2.8	<0.0001	1.75-4.3
LN status positive	2.5	0.0049	1.3-4.7	3.3	<0.0001	2.3-4.9
less than 55 y.o.	1.2	0.65	0.62-2.1	1.1	0.70	0.74-1.5
TNBC	3.3	0.0010	1.7-6.2	1.7	0.039	1.0-2.6

Supplementary Table 5. Genes for targeted sequencing of tumor samples

*AKT1**	*PTEN**	*BRAF*	*FANCA*	*MYC*	*SMARCD1*
*ARID1A**	*RB1**	*BRIP1*	*FGFR1*	*NBN*	*SRPR*
*BRCA1**	*RUNX1**	*CCND1*	*FGFR2*	*NCOR2*	*STK11*
*BRCA2**	*SF3B1**	*CCND2*	*FGFR3*	*NRAS*	*SYNE2*
*CASP8**	*TBX3**	*CCND3*	*FGFR4*	*OR2G3*	*TBL1XR1*
*CBFB**	*TP53**	*CCNE1*	*FOXO3*	*PAK1*	*TBX4*
*CDH1**	*XBP1**	*CDK4*	*GPS2*	*PALB2*	*TBX5*
*CDKN1B**	*ZFP36L1**	*CDK6*	*IGF1R*	*PDGFRA*	*TRIM6-TRIM34*
*CTCF**	*CDKN2A***	*CDKN2C*	*IKBKB*	*PIWIL1*	*TTN*
*FOXA1**	*KRAS***	*CHD1*	*INPP4B*	*PNPLA3*	*UBR5*
*GATA3**	*AFF2*	*CHEK2*	*JAK2*	*PTPN22*	*USH2A*
*MAP2K4**	*AKT2*	*CSMD1*	*KCNB2*	*PTPRD*	*WNT7A*
*MAP3K1**	*AKT3*	*DCAF4L2*	*KDM3A*	*RAD51C*	*WWOX*
*MED23**	*APOBEC3A*	*DGKG*	*KIT*	*RECQL*	*ZNF217*
*MLL3 (KMT2C)**	*APOBEC3B*	*EGFR*	*MAGI3*	*RPGR*	*ZNF703*
*MYB**	*ATM*	*ERBB2*	*MAP3K13*	*RSF1*	
*NCOR1**	*ATN1*	*ERBB3*	*MCL1*	*RYR2*	
*NF1**	*ATP2B2*	*ERRB*	*MDM2*	*SETD1A*	
*PIK3CA**	*ATR*	*ESR1*	*MET*	*SETD2*	
*PIK3R1**	*BCL2L1*	*FAM47C*	*MLL2 (KMT2D)*	*SMAD4*	

* indicates 28 driver genes reported in Nik-Zainel et al., ***Nature.*** 2016.

**indicates two genes with driver mutations reported in COSMIC database.

Supplementary Table 6. Driver mutations identified in 60 breast tumors

Sample	Germline	Biallelic inactivatio	Chr	Start	End	Ref	Alt	Gene	Exonic Function	Amino acid change	VAF
KU088	*BRCA2*	-	7	1.52E+08	1.52E+08	G	-	*KMT2C*	Nonsense	KMT2C:NM_170606:exon14:c.2448delC:p.Y816X	0.048
KU088	*BRCA2*	-	10	8111543	8111545	CTA	-	*GATA3*	Inframe indel	TA3:NM_001002295:exon5:c.1032_1034del:p.344_345	0.185
KU088	*BRCA2*	-	10	89720805	89720808	CTTT	-	*PTEN*	Frameshift indel	PTEN:NM_000314:exon8:c.956_959del:p.T319fs	0.253
KU088	*BRCA2*	-	16	68867262	68867262	G	T	*CDH1*	Nonsense	CDH1:NM_004360:exon16:c.G2509T:p.G837X	0.349
KU089	*BRCA2*	+	5	67593395	67593395	T	A	*PIK3R1*	Missense	PIK3R1:NM_181523:exon16:c.T2141A:p.L714Q	0.356
KU089	*BRCA2*	+	13	32954156	32954160	ATTTT	-	*BRCA2*	Frameshift indel	BRCA2:NM_000059:exon24:c.9130_9134del:p.I3044fs	0.074
KU085	*BRCA1*	+	17	7578213	7578214	AA	-	*TP53*	Frameshift indel	TP53:NM_000546:exon6:c.635_636del:p.F212fs	0.624
KU048	None	NA	17	7579363	7579363	-	C	*TP53*	Frameshift indel	TP53:NM_000546:exon4:c.323dupG:p.G108fs	0.435
KU049	None	NA	3	1.79E+08	1.79E+08	A	G	*PIK3CA*	Missense	PIK3CA:NM_006218:exon21:c.A3140G:p.H1047R	0.407
KU050	None	NA	10	8111433	8111434	CA	-	*GATA3*	Splice site mutation	GATA3:NM_001002295:exon5:c.925-3_925-1del	0.276
KU050	None	NA	10	89624272	89624272	-	A	*PTEN*	Nonsense	PTEN:NM_000314:exon1:c.47dupA:p.Y16_Q17delinsX	0.273
KU050	None	NA	10	89692905	89692905	G	A	*PTEN*	Missense	PTEN:NM_000314:exon5:c.G389A:p.R130Q	0.309
KU052	None	NA	7	1.52E+08	1.52E+08	C	A	*KMT2C*	Nonsense	KMT2C:NM_170606:exon52:c.G13141T:p.E4381X	0.291
KU052	None	NA	7	1.52E+08	1.52E+08	G	A	*KMT2C*	Missense	KMT2C:NM_170606:exon15:c.C2633T:p.A878V	0.037
KU052	None	NA	10	89685309	89685309	C	A	*PTEN*	Nonsense	PTEN:NM_000314:exon3:c.C204A:p.Y68X	0.263
KU077	None	NA	3	1.79E+08	1.79E+08	A	G	*PIK3CA*	Missense	PIK3CA:NM_006218:exon21:c.A3140G:p.H1047R	0.424
KU077	None	NA	17	7578550	7578550	G	A	*TP53*	Missense	TP53:NM_000546:exon5:c.C380T:p.S127F	0.383
KU077	None	NA	17	41256906	41256906	G	A	*BRCA1*	Nonsense	BRCA1:NM_007294:exon5:c.C280T:p.Q94X	0.388
KU053	None	NA	3	1.79E+08	1.79E+08	A	G	*PIK3CA*	Missense	PIK3CA:NM_006218:exon21:c.A3140G:p.H1047R	0.183
KU053	None	NA	14	1.05E+08	1.05E+08	G	T	*AKT1*	Missense	AKT1:NM_005163:exon4:c.C235A:p.Q79K	0.344
KU054	None	NA	3	1.79E+08	1.79E+08	G	A	*PIK3CA*	Missense	PIK3CA:NM_006218:exon10:c.G1633A:p.E545K	0.23
KU054	None	NA	3	1.79E+08	1.79E+08	G	A	*PIK3CA*	Missense	PIK3CA:NM_006218:exon14:c.G2176A:p.E726K	0.058
KU054	None	NA	12	1.15E+08	1.15E+08	C	-	*TBX3*	Frameshift indel	TBX3:NM_005996:exon1:c.359delG:p.G120fs	0.251
KU054	None	NA	14	38061463	38061463	T	C	*FOXA1*	Missense	FOXA1:NM_004496:exon2:c.A526G:p.I176V	0.265
KU054	None	NA	16	68772218	68772218	C	T	*CDH1*	Nonsense	CDH1:NM_004360:exon2:c.C67T:p.Q23X	0.41
KU055	None	NA	10	89624307	89624307	T	-	*PTEN*	Splice site mutation	PTEN:,NM_000314:exon1:c.79+2T>-	0.03
KU056	None	NA	5	56155659	56155659	C	-	*MAP3K1*	Frameshift indel	MAP3K1:NM_005921:exon3:c.751delC:p.P251fs	0.081
KU056	None	NA	6	1.36E+08	1.36E+08	-	G	*MYB*	Frameshift indel	MYB:NM_001130173:exon7:c.822dupG:p.Q274fs	0.052
KU078	None	NA	3	1.79E+08	1.79E+08	T	A	*PIK3CA*	Missense	PIK3CA:NM_006218:exon5:c.T1035A:p.N345K	0.333
KU078	None	NA	10	8111433	8111434	CA	-	*GATA3*	Splice site mutation	GATA3:NM_001002295:exon5:c.925-3_925-1del	0.348
KU078	None	NA	12	12870841	12870841	-	C	*CDKN1B*	Frameshift indel	CDKN1B:NM_004064:exon1:c.69dupC:p.H23fs	0.069
KU078	None	NA	16	67644810	67644810	C	A	*CTCF*	Nonsense	CTCF:NM_006565:exon3:c.C75A:p.Y25X	0.433
KU057	None	NA	3	1.79E+08	1.79E+08	A	G	*PIK3CA*	Missense	PIK3CA:NM_006218:exon21:c.A3140G:p.H1047R	0.224
KU057	None	NA	12	1.15E+08	1.15E+08	-	T	*TBX3*	Nonsense	TBX3:NM_005996:exon3:c.683dupA:p.Y228_Q229delins	0.232
KU057	None	NA	16	67663341	67663341	-	G	*CTCF*	Frameshift indel	CTCF:NM_006565:exon10:c.1742_1743insG:p.D581fs	0.204
KU057	None	NA	17	12032546	12032546	C	G	*MAP2K4*	Missense	MAP2K4:NM_003010:exon9:c.C982G:p.L328V	0.294
KU057	None	NA	17	16012220	16012220	G	A	*NCOR1*	Nonsense	NCOR1:NM_006311:exon19:c.C2062T:p.R688X	0.276
KU058	None	NA	3	1.79E+08	1.79E+08	T	A	*PIK3CA*	Missense	PIK3CA:NM_006218:exon5:c.T1035A:p.N345K	0.17
KU059	None	NA	17	29541543	29541543	T	G	*NF1*	Nonsense	NF1:NM_000267:exon13:c.T1467G:p.Y489X	0.691
KU062	None	NA	5	67589595	67589618	TTCAAG	-	*PIK3R1*	Inframe indel	IK3R1:NM_181523:exon11:c.1358_1381del:p.453_461d	0.108
KU062	None	NA	9	21971111	21971111	G	A	*CDKN2A*	Missense	CDKN2A:NM_000077:exon2:c.C247T:p.H83Y	0.257
KU062	None	NA	17	7578222	7578223	TC	-	*TP53*	Frameshift indel	TP53:NM_000546:exon6:c.626_627del:p.R209fs	0.263
KU062	None	NA	17	29490394	29490394	G	C	*NF1*	Missense	NF1:NM_000267:exon4:c.G479C:p.R160T	0.184
KU062	None	NA	17	29665804	29665804	T	G	*NF1*	Nonsense	NF1:NM_000267:exon45:c.T6839G:p.L2280X	0.193
KU063	None	NA	3	1.79E+08	1.79E+08	A	G	*PIK3CA*	Missense	PIK3CA:NM_006218:exon21:c.A3140G:p.H1047R	0.199
KU064	None	NA	3	1.79E+08	1.79E+08	A	G	*PIK3CA*	Missense	PIK3CA:NM_006218:exon21:c.A3140G:p.H1047R	0.58
KU064	None	NA	3	1.79E+08	1.79E+08	A	G	*PIK3CA*	Missense	PIK3CA:NM_006218:exon21:c.A3143G:p.H1048R	0.571
KU064	None	NA	13	32913908	32913908	G	A	*BRCA2*	Missense	BRCA2:NM_000059:exon11:c.G5416A:p.E1806K	0.211
KU064	None	NA	17	7579699	7579699	C	G	*TP53*	Splice site mutation	TP53:NM_000546:exon3:c.C96+1G>C	0.389
KU065	None	NA	10	8111433	8111434	CA	-	*GATA3*	Splice site mutation	GATA3:NM_001002295:exon5:c.925-3_925-1del	0.227
KU065	None	NA	16	68853266	68853266	G	C	*CDH1*	Missense	CDH1:NM_004360:exon11:c.G1649C:p.R550T	0.275
KU065	None	NA	21	36259192	36259192	G	A	*RUNX1*	Missense	RUNX1:NM_001754:exon4:c.C299T:p.S100F	0.315
KU014	*BRCA1*	-	17	7577529	7577529	A	T	*TP53*	Missense	TP53:NM_000546:exon7:c.T752A:p.I251N	0.072
KU014	*BRCA1*	-	17	16004716	16004716	C	G	*NCOR1*	Missense	NCOR1:NM_006311:exon20:c.G2538C:p.L846F	0.054
KU014	*BRCA1*	-	17	29683498	29683498	C	T	*NF1*	Missense	NF1:NM_000267:exon51:c.C7573T:p.H2525Y	0.046
KU016	*BRCA1*	-	3	1.79E+08	1.79E+08	A	G	*PIK3CA*	Missense	PIK3CA:NM_006218:exon21:c.A3140G:p.H1047R	0.573
KU067	None	NA	21	36206869	36206869	-	G	*RUNX1*	Frameshift indel	RUNX1:NM_001754:exon7:c.642dupC:p.K215fs	0.052
KU079	None	NA	7	1.52E+08	1.52E+08	AGCTGG	-	*KMT2C*	Frameshift indel	KMT2C:NM_170606:exon35:c.5175_5190del:p.I1725fs	0.033
KU079	None	NA	10	8111433	8111434	CA	-	*GATA3*	Splice site mutation	GATA3:NM_001002295:exon5:c.925-3_925-1del	0.515
KU079	None	NA	10	89624243	89624243	A	T	*PTEN*	Missense	PTEN:NM_000314:exon1:c.A17T:p.K6I	0.122
KU079	None	NA	14	38061227	38061227	G	C	*FOXA1*	Missense	FOXA1:NM_004496:exon2:c.C762G:p.F254L	0.298
KU079	None	NA	14	38061240	38061240	G	A	*FOXA1*	Missense	FOXA1:NM_004496:exon2:c.C749T:p.S250F	0.498
KU068	None	NA	3	1.79E+08	1.79E+08	A	T	*PIK3CA*	Missense	PIK3CA:NM_006218:exon5:c.A823T:p.S275C	0.049
KU068	None	NA	3	1.79E+08	1.79E+08	A	G	*PIK3CA*	Missense	PIK3CA:NM_006218:exon21:c.A3140G:p.H1047R	0.282
KU068	None	NA	7	1.52E+08	1.52E+08	T	A	*KMT2C*	Missense	KMT2C:NM_170606:exon50:c.A12606T:p.K4202N	0.065
KU068	None	NA	14	69256499	69256502	CTGG	-	*ZFP36L1*	Frameshift indel	ZFP36L1:NM_004926:exon2:c.765_768del:p.S255fs	0.047
KU068	None	NA	16	68842394	68842395	AG	-	*CDH1*	Frameshift indel	CDH1:NM_004360:exon4:c.455_456del:p.Q152fs	0.053
KU068	None	NA	17	7577564	7577564	-	T	*TP53*	Frameshift indel	TP53:NM_000546:exon7:c.716dupA:p.N239fs	0.414
KU071	None	NA	5	56178376	56178376	G	T	*MAP3K1*	Nonsense	MAP3K1:NM_005921:exon14:c.G3349T:p.E1117X	0.141
KU072	None	NA	10	89624242	89624243	AA	-	*PTEN*	Frameshift indel	PTEN:NM_000314:exon1:c.16_17del:p.K6fs	0.422
KU072	None	NA	14	38061167	38061167	G	-	*FOXA1*	Frameshift indel	FOXA1:NM_004496:exon2:c.822delC:p.A274fs	0.263
KU080	None	NA	3	1.79E+08	1.79E+08	C	G	*PIK3CA*	Missense	PIK3CA:NM_006218:exon10:c.C1616G:p.P539R	0.223
KU080	None	NA	12	1.15E+08	1.15E+08	-	A	*TBX3*	Frameshift indel	TBX3:NM_005996:exon3:c.782dupT:p.V261fs	0.109
KU080	None	NA	17	7577120	7577120	C	T	*TP53*	Missense	TP53:NM_000546:exon8:c.G818A:p.R273H	0.201
KU081	None	NA	5	67589585	67589587	CAT	-	*PIK3R1*	Inframe indel	IK3R1:NM_181523:exon11:c.1348_1350del:p.450_450d	0.294
KU081	None	NA	10	89711874	89711874	G	A	*PTEN*	Splice site mutation	PTEN:NM_000314:exon6:c.493-1G>A	0.174
KU081	None	NA	10	89720855	89720855	-	A	*PTEN*	Nonsense	TEN:NM_000314:exon8:c.1007dupA:p.Y336_F337delins	0.187
KU081	None	NA	17	7578442	7578442	T	C	*TP53*	Missense	TP53:NM_000546:exon5:c.A488G:p.Y163C	0.2
KU081	None	NA	17	7579533	7579533	G	A	*TP53*	Nonsense	TP53:NM_000546:exon4:c.C154T:p.Q52X	0.765
KU082	None	NA	17	29559112	29559112	G	T	*NF1*	Missense	NF1:NM_000267:exon25:c.G3219T:p.M1073I	0.078
KU073	None	NA	3	1.79E+08	1.79E+08	A	G	*PIK3CA*	Missense	PIK3CA:NM_006218:exon21:c.A3140G:p.H1047R	0.135
KU019	*BRCA2*	-	10	89717696	89717697	TT	-	*PTEN*	Frameshift indel	PTEN:NM_000314:exon7:c.721_722del:p.F241fs	0.087
KU021	*BRCA2*	+	10	8115874	8115874	-	G	*GATA3*	Frameshift indel	GATA3:NM_001002295:exon6:c.1224dupG:p.S408fs	0.242
KU003	*BRCA2*	-	7	1.52E+08	1.52E+08	C	-	*KMT2C*	Frameshift indel	KMT2C:NM_170606:exon54:c.14075delG:p.G4692fs	0.243
KU003	*BRCA2*	-	7	1.52E+08	1.52E+08	G	-	*KMT2C*	Frameshift indel	KMT2C:NM_170606:exon14:c.2024delC:p.T675fs	0.308
KU003	*BRCA2*	-	12	25398284	25398284	C	G	*KRAS*	Missense	KRAS:NM_033360:exon2:c.G35C:p.G12A	0.104
KU074	None	NA	3	1.79E+08	1.79E+08	A	G	*PIK3CA*	Missense	PIK3CA:NM_006218:exon21:c.A3140G:p.H1047R	0.21
KU074	None	NA	17	7577120	7577120	C	A	*TP53*	Missense	TP53:NM_000546:exon8:c.G818T:p.R273L	0.246
KU075	None	NA	3	1.79E+08	1.79E+08	G	A	*PIK3CA*	Missense	PIK3CA:NM_006218:exon2:c.G263A:p.R88Q	0.131
KU075	None	NA	3	1.79E+08	1.79E+08	G	A	*PIK3CA*	Missense	PIK3CA:NM_006218:exon10:c.G1633A:p.E545K	0.083
KU075	None	NA	7	1.52E+08	1.52E+08	C	-	*KMT2C*	Frameshift indel	KMT2C:NM_170606:exon8:c.1139delG:p.R380fs	0.039
KU075	None	NA	10	8111433	8111434	CA	-	*GATA3*	Splice site mutation	GATA3:NM_001002295:exon5:c.925-3_925-1del	0.125
KU075	None	NA	14	38061259	38061259	A	G	*FOXA1*	Missense	FOXA1:NM_004496:exon2:c.T730C:p.W244R	0.16
KU075	None	NA	16	67070560	67070562	ACC	-	*CBFB*	Inframe indel	CBFB:NM_022845:exon3:c.184_186del:p.62_62del	0.171
KU075	None	NA	16	68842656	68842656	-	AC	*CDH1*	Frameshift indel	CDH1:NM_004360:exon5:c.592_593insAC:p.D198fs	0.134
KU022	*BRCA2*	+	12	12871754	12871761	CTTAGAT	-	*CDKN1B*	Inframe indel	CDKN1B:NM_004064:exon2:c.476_478del:p.159_160de	0.21
KU022	*BRCA2*	+	13	49039379	49039400	AGTTTC	-	*RB1*	Frameshift indel	RB1:NM_000321:exon23:c.2364_2385del:p.S788fs	0.143
KU005	*BRCA2*	-	2	1.98E+08	1.98E+08	C	A	*SF3B1*	Missense	SF3B1:NM_012433:exon2:c.G163T:p.V55L	0.097
KU005	*BRCA2*	-	3	1.79E+08	1.79E+08	G	T	*PIK3CA*	Missense	PIK3CA:NM_006218:exon21:c.G3129T:p.M1043I	0.115
KU076	None	NA	3	1.79E+08	1.79E+08	G	A	*PIK3CA*	Missense	PIK3CA:NM_006218:exon10:c.G1633A:p.E545K	0.51
KU007	*BRCA2*	+	6	1.32E+08	1.32E+08	T	A	*MED23*	Missense	MED23:NM_004830:exon12:c.A1158T:p.K386N	0.209

KU033	*BRCA2*	+	7	1.52E+08	1.52E+08	C	T	*KMT2C*	Missense	KMT2C:NM_170606:exon51:c.G12674A:p.R4225Q	0.291
KU033	*BRCA2*	+	17	7578212	7578212	G	A	*TP53*	Nonsense	TP53:NM_000546:exon6:c.C637T:p.R213X	0.734
KU034	*BRCA2*	-	3	1.79E+08	1.79E+08	A	G	*PIK3CA*	Missense	PIK3CA:NM_006218:exon21:c.A3140G:p.H1047R	0.035
KU035	*BRCA2*	-	3	1.79E+08	1.79E+08	A	G	*PIK3CA*	Missense	PIK3CA:NM_006218:exon21:c.A3140G:p.H1047R	0.401
KU035	*BRCA2*	-	17	7577506	7577506	C	A	*TP53*	Missense	TP53:NM_000546:exon7:c.G775T:p.D259Y	0.13
KU035	*BRCA2*	-	17	11984782	11984782	C	T	*MAP2K4*	Nonsense	MAP2K4:NM_003010:exon3:c.C328T:p.R110X	0.155
KU038	*BRCA2*	-	3	1.79E+08	1.79E+08	G	A	*PIK3CA*	Missense	PIK3CA:NM_006218:exon2:c.G241A:p.E81K	0.612
KU038	*BRCA2*	-	3	1.79E+08	1.79E+08	A	G	*PIK3CA*	Missense	PIK3CA:NM_006218:exon21:c.A3140G:p.H1047R	0.639
KU038	*BRCA2*	-	5	56161243	56161243	C	G	*MAP3K1*	Nonsense	MAP3K1:NM_005921:exon5:c.C1112G:p.S371X	0.645
KU038	*BRCA2*	-	6	1.36E+08	1.36E+08	T	G	*MYB*	Missense	MYB:NM_001130173:exon10:c.T1489G:p.S497A	0.348
KU038	*BRCA2*	-	7	1.52E+08	1.52E+08	G	A	*KMT2C*	Missense	KMT2C:NM_170606:exon15:c.C2633T:p.A878V	0.11
KU038	*BRCA2*	-	12	25398284	25398284	C	T	*KRAS*	Missense	KRAS:NM_033360:exon2:c.G35A:p.G12D	0.436
KU041	*BRCA2*	+	5	67522729	67522729	-	A	*PIK3R1*	Nonsense	PIK3R1:NM_181523:exon2:c.227dupA:p.Y76_I77delinsX	0.22
KU041	*BRCA2*	+	7	1.52E+08	1.52E+08	T	C	*KMT2C*	Missense	KMT2C:NM_170606:exon44:c.A11650G:p.T3884A	0.048
KU041	*BRCA2*	+	7	1.52E+08	1.52E+08	T	A	*KMT2C*	Missense	KMT2C:NM_170606:exon8:c.A1167T:p.K389N	0.027
KU041	*BRCA2*	+	7	1.52E+08	1.52E+08	C	A	*KMT2C*	Missense	KMT2C:NM_170606:exon7:c.G944T:p.G315V	0.058
KU043	*BRCA2*	+	3	1.79E+08	1.79E+08	A	G	*PIK3CA*	Missense	PIK3CA:NM_006218:exon21:c.A3140G:p.H1047R	0.489
KU043	*BRCA2*	+	16	68847295	68847309	[illegible]GCGTG[illegible]	-	*CDH1*	Inframe indel	CDH1:NM_004360:exon9:c.1217_1231del:p.406_411del	0.078
KU045	*BRCA1*	+	7	1.52E+08	1.52E+08	G	T	*KMT2C*	Missense	KMT2C:NM_170606:exon8:c.C1179A:p.N393K	0.033
KU045	*BRCA1*	+	17	7577031	7577031	T	-	*TP53*	Frameshift indel	TP53:NM_000546:exon8:c.907delA:p.S303fs	0.626
KU009	*BRCA1*	+	17	7578484	7578484	-	A	*TP53*	Frameshift indel	TP53:NM_000546:exon5:c.445dupT:p.S149fs	0.358

Supplementary Table 7. The landscape of driver mutations in patients who have germline *BRCA1/2* variants.

Patient	Germline variant	LOH	Somatic variant
KU043	*BRCA2*	del	*PIK3CA, CDH1*
KU033	*BRCA2*	del	*TP53, KMT2C*
KU041	*BRCA2*	del	*KMT2C, PIK3R1*
KU021	*BRCA2*	del	*GATA3*
KU012	*BRCA2*	del	wt
KU089	*BRCA2*	del	*PIK3R1*
KU042	*BRCA2*	del	wt
KU037	*BRCA2*	del	wt
KU007	*BRCA2*	del	wt
KU006	*BRCA2*	del	wt
KU032	*BRCA2*	del	wt
KU028	*BRCA2*	del	wt
KU022	*BRCA2*	del	wt
KU011	*BRCA2*	del	wt
KU084	*BRCA2*	del	wt
KU091	*BRCA2*	del	wt
KU087	*BRCA2*	del	wt
KU038	*BRCA2*	wt	*PIK3CA, KMT2C, KRAS*
KU035	*BRCA2*	wt	*PIK3CA, TP53*
KU034	*BRCA2*	wt	*PIK3CA*
KU005	*BRCA2*	wt	*PIK3CA*
KU003	*BRCA2*	wt	*KMT2C, KRAS*
KU088	*BRCA2*	wt	*KMT2C, CDH1, GATA3,PTEN*
KU019	*BRCA2*	wt	*PTEN*
KU083	*BRCA2*	wt	wt
KU045	*BRCA1*	amp	*TP53, KMT2C*
KU009	*BRCA1*	del	*TP53*
KU085	*BRCA1*	UPD	*TP53*
KU016	*BRCA1*	wt	*PIK3CA*
KU014	*BRCA1*	wt	*TP53*

Abbreviations: del, deletion ; amp, amplification ; UPD, uniparental disomy ; wt, wild type

Supplementary Table 8. Clinical correlates with *BRCA1/2* biallelic inactivation

	BRCA biallelic inactivation				Non *BRCA*		Without germline variants		*P* value		
	Present		Absent						Biallelic inactivation present vs. absent	Biallelic inactivation present vs. without germline variants	Biallelic inactivation absent vs. without germline variants
N	49		15		24		1,894				
	N	%	N	%	N	%	N	%			
Avg Age diagnosis (±SD)	48.7±13.7		57.9±12.1		54.4 ± 14.9		59.0±12.8		0.0137	<0.0001	0.6055
T											
is, 1	13	26.5	7	46.7	12	50.0	1071	56.5			
2,3,4	32	65.3	5	33.3	10	41.7	799	42.2	0.0884	0.0002	1.0000
N											
0	21	42.9	8	53.3	14	58.3	1463	77.2			
1,2,3	24	49.0	4	26.7	8	33.3	398	21.0	0.3313	0.0001	0.2997
Subtype											
non TNBC	31	63.3	12	80.0	16	66.7	1477	77.2			
TNBC	13	26.5	3	20.0	3	12.5	200	10.6	0.7376	0.0017	0.4107
Deceased	4	8.2	1	6.7	0	0.0	50	2.6	1.0000	0.0448	0.3348

Supplementary Table 9. Multivariate analysis of survival with biallelic inactivation of *BRCA1/2* as a covariate

	OS			DFS		
	Hazard ratio	P-value	95% CI	Hazard ratio	P-value	95% CI
LOH positive	2.10E-09	1	0.0-1.2	0.38	0.12	0.062-1.2
LOH negative	1.5	0.67	0.2-11	1.8	0.36	0.44-4.8
larger than T3	3.2	0.0022	1.5-6.7	3.0	<0.0001	1.9-4.7
LN status positive	2.4	0.0063	1.3-4.6	3.4	<0.0001	2.3-5.0
less than 55 y.o.	0.88	0.68	0.47-1.6	0.91	0.63	0.63-1.3
TNBC	3.4	0.0002	1.8-6.6	1.7	0.031	1.1-2.7

Supplementary Figure 1

a

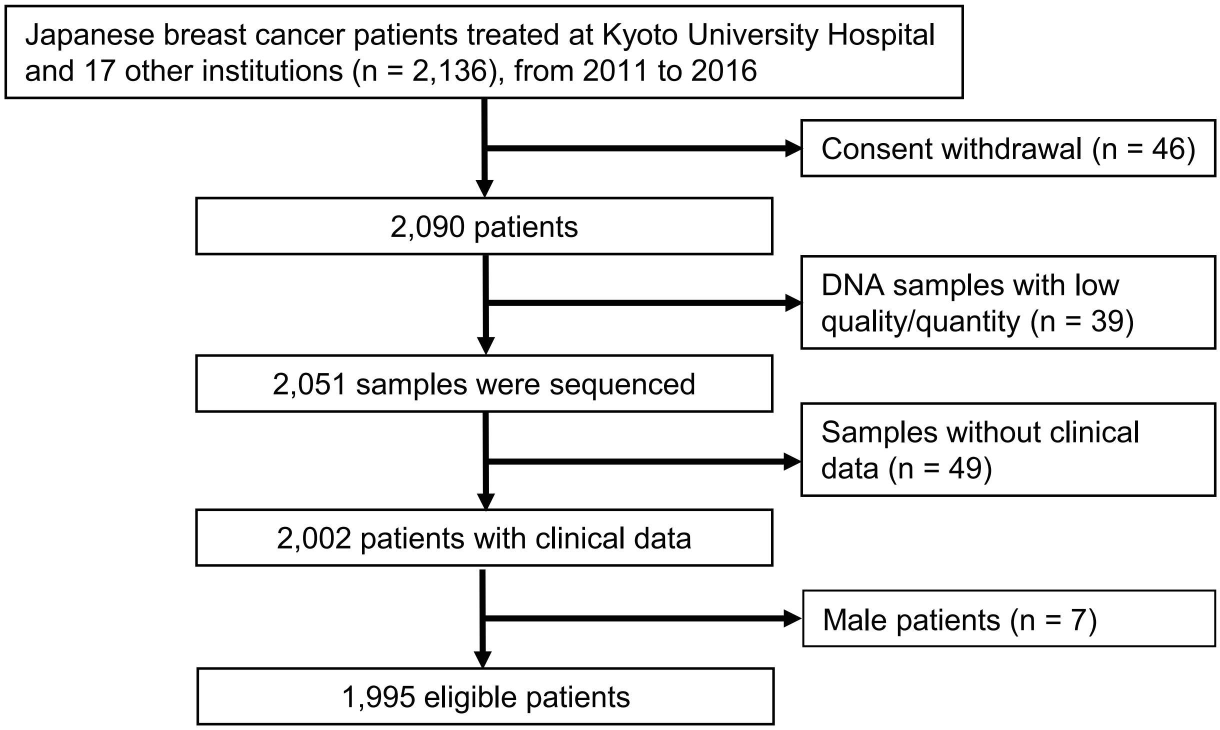

b

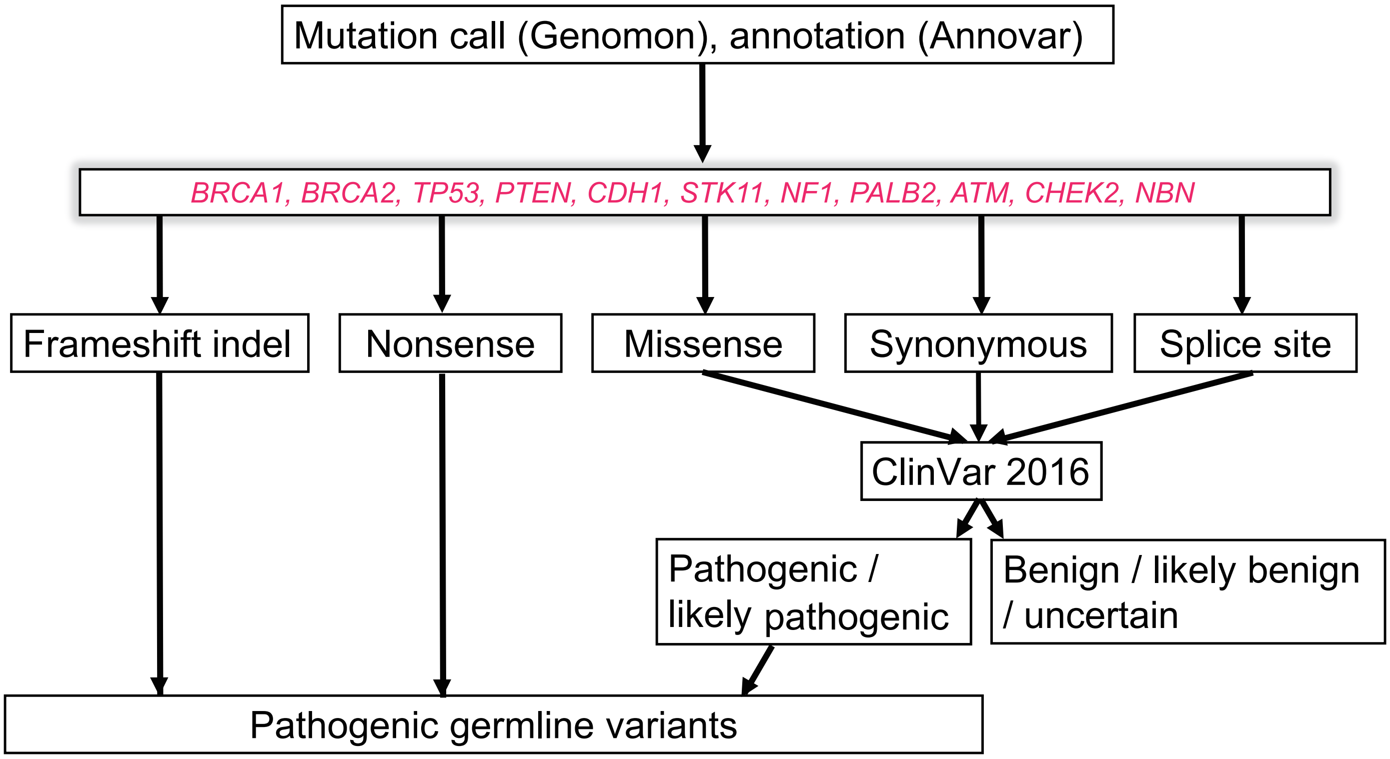

Supplementary Figure 2

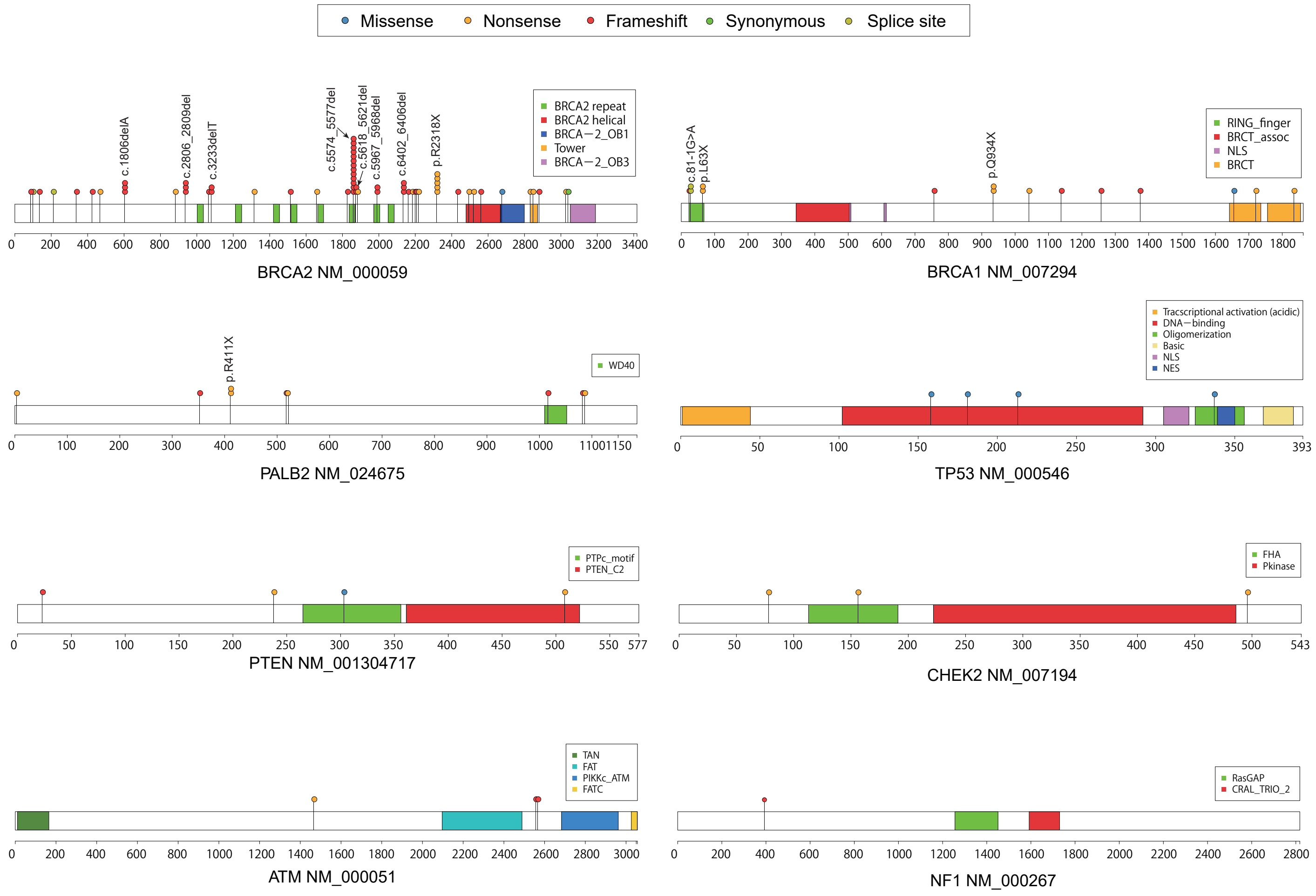

Supplementary Figure 3

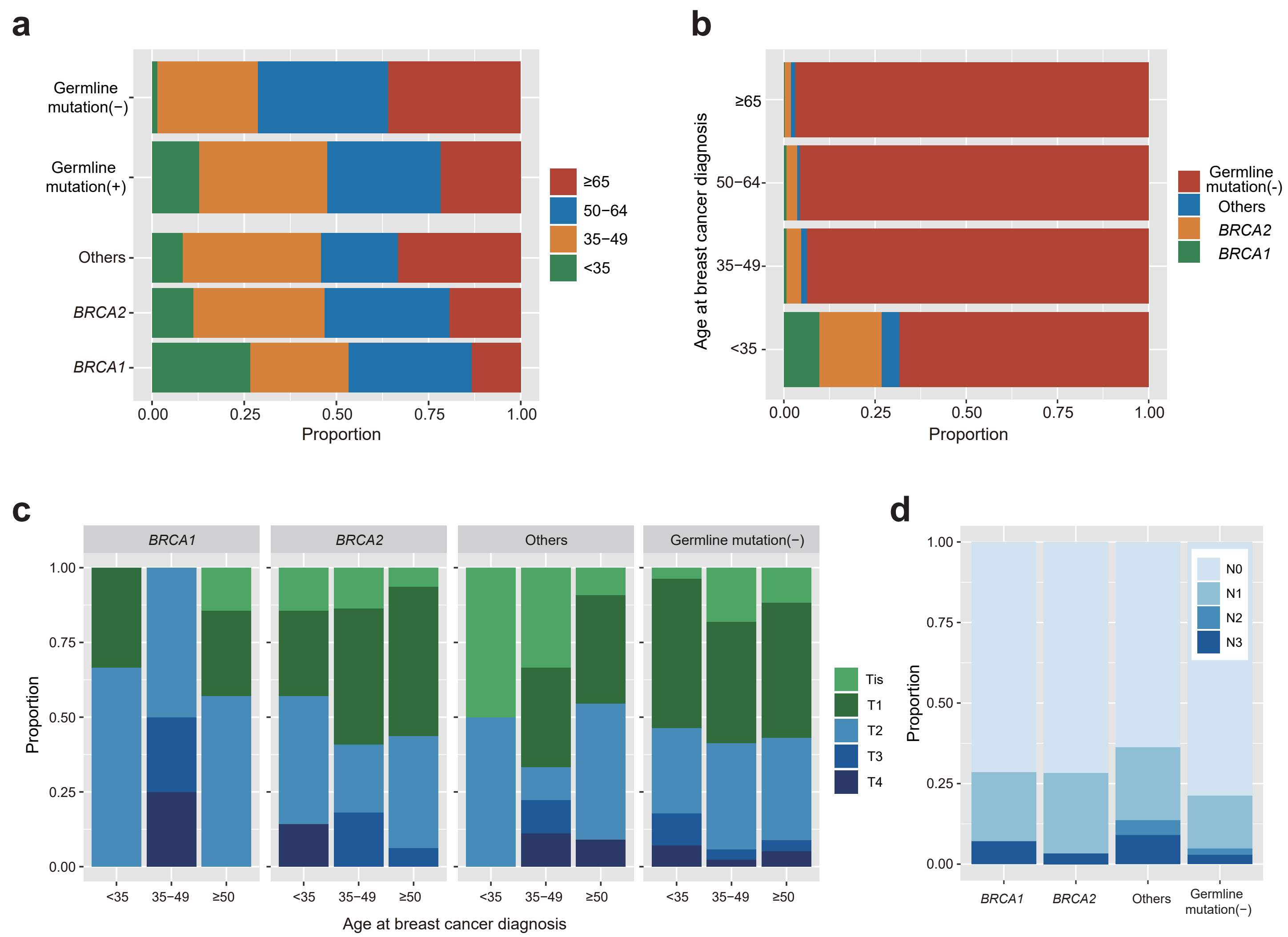

Supplementary Figure 4

a

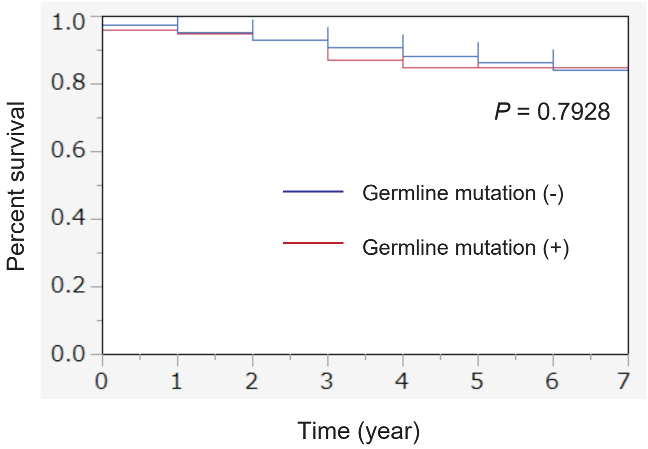

b

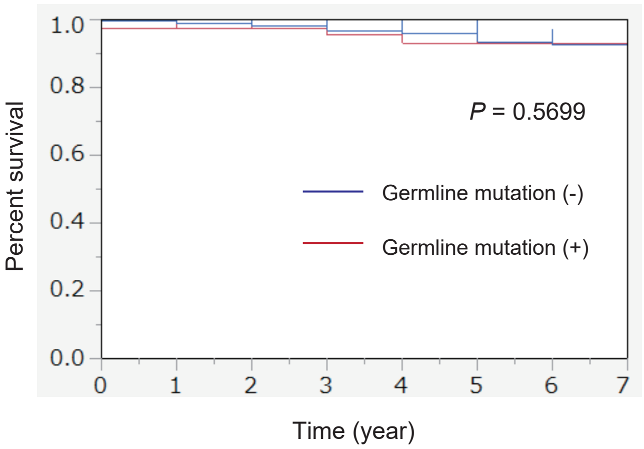

Supplementary Figure 5

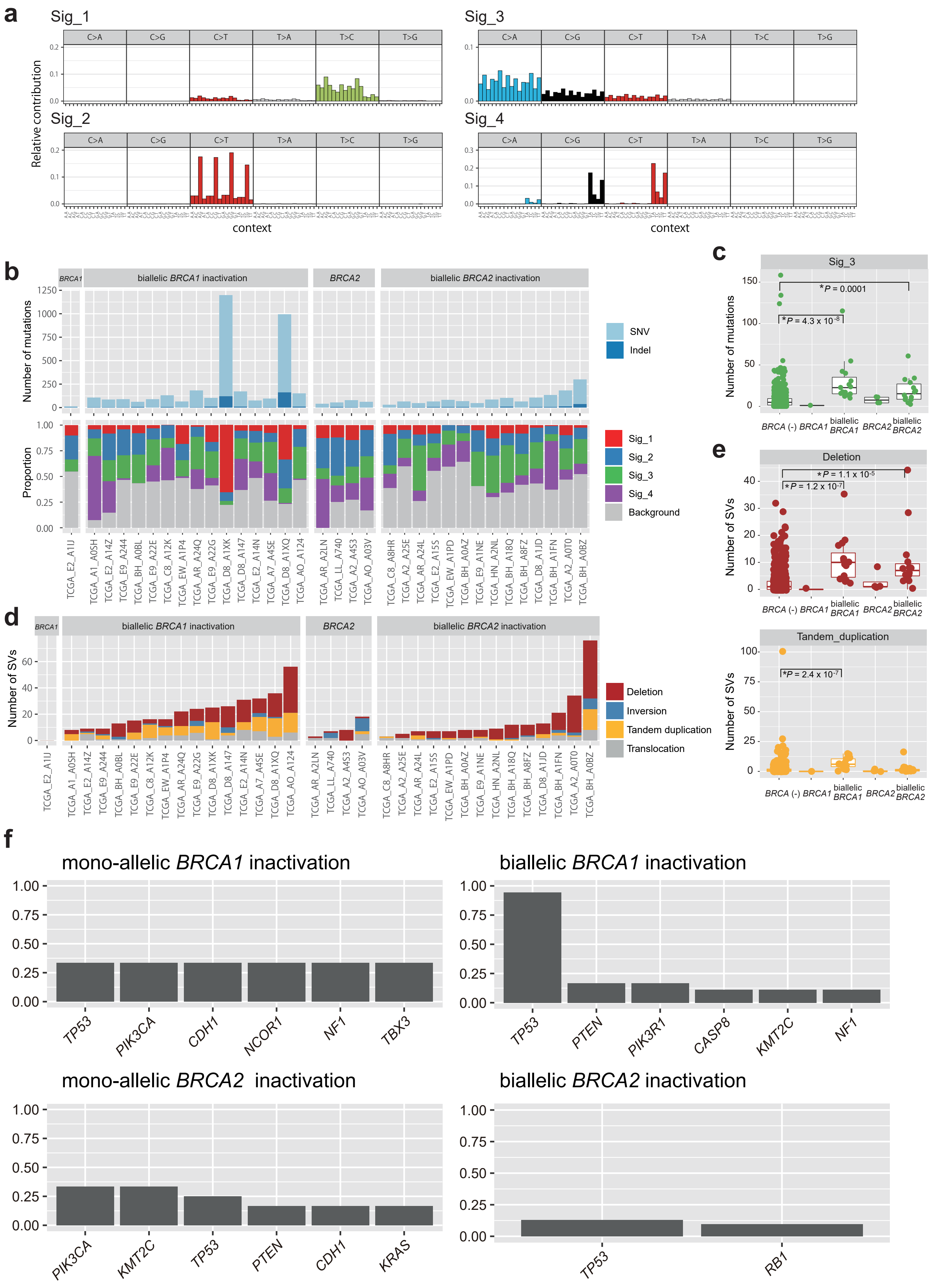

Supplementary Figure 6

a

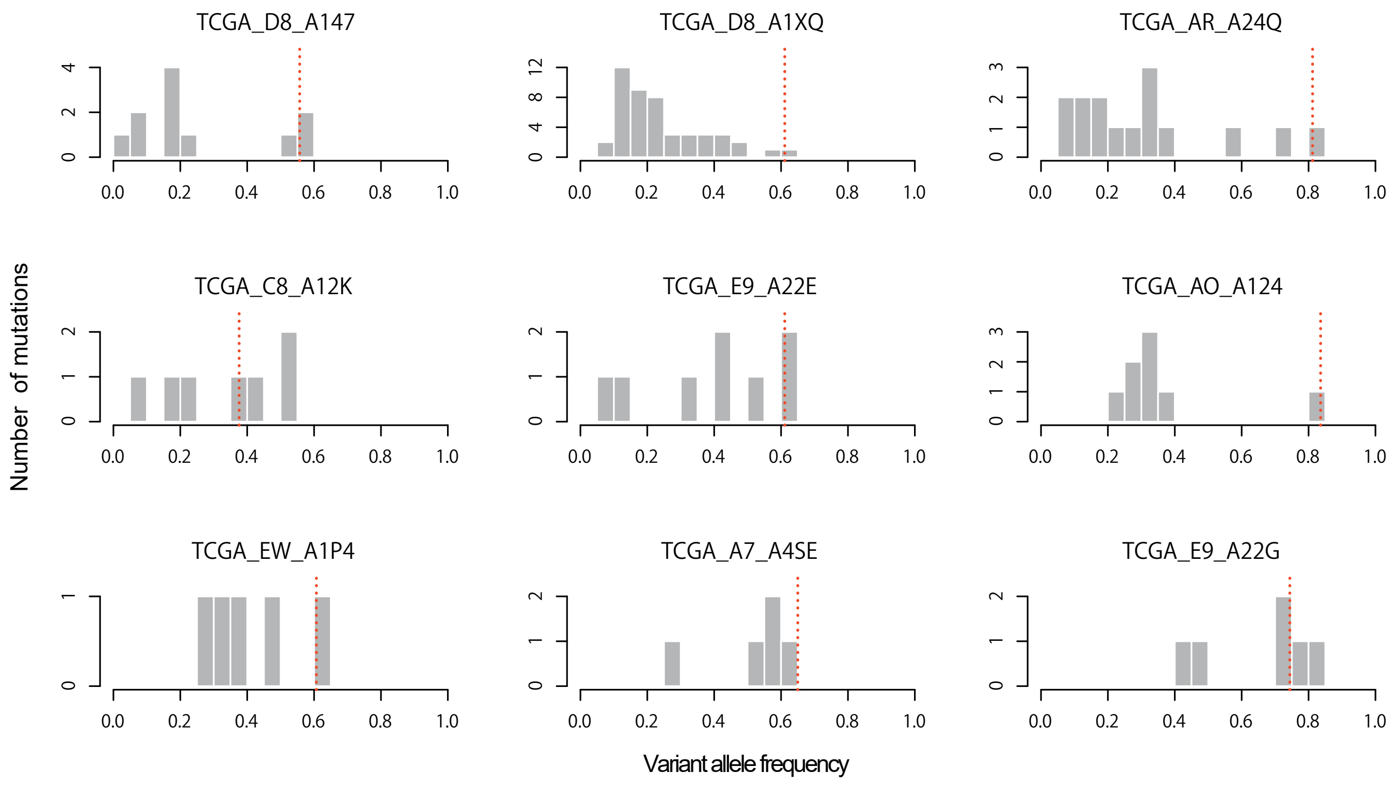

b

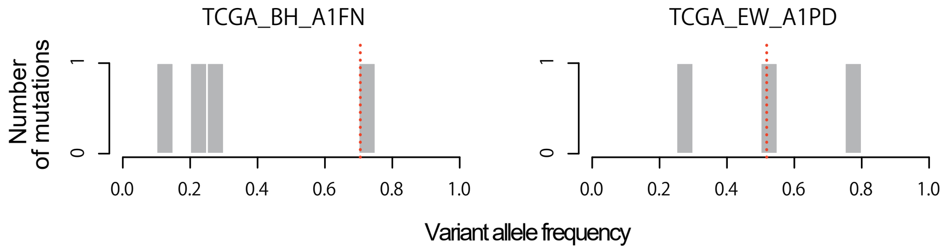

Supplementary Figure 7

a

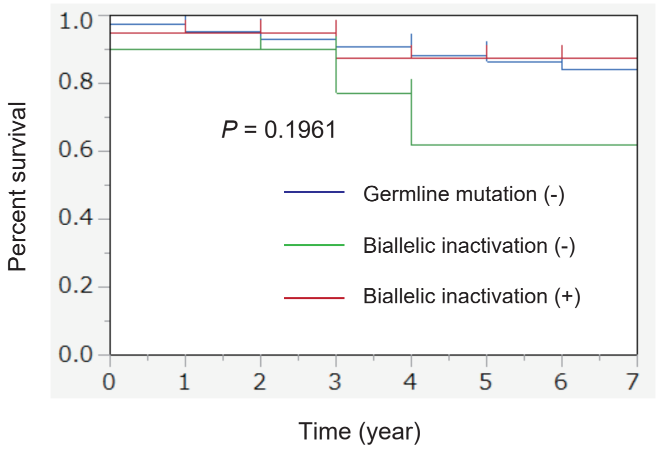

b

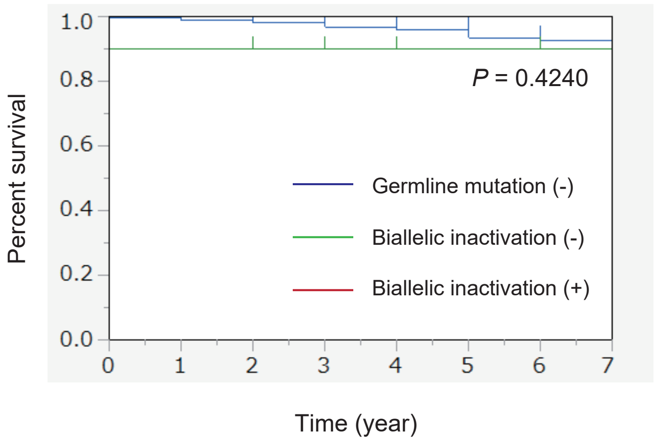

Supplementary Figure 8

Whole genome amplification (n = 9)
TCGA-A2-A0D2
TCGA-A8-A09A
TCGA-A8-A097
TCGA-A8-A09W
TCGA-B6-A0RG
TCGA-BH-A0B4
TCGA-AN-A0AL
TCGA-AN-A0FL
TCGA-AN-A0FX

Low quality of copy number data (n = 3)
TCGA_AC_A3BB
TCGA_AR_A1AM
TCGA_E9_A3QA

Low tumor purity (n = 1)
TCGA_EW_A1P7

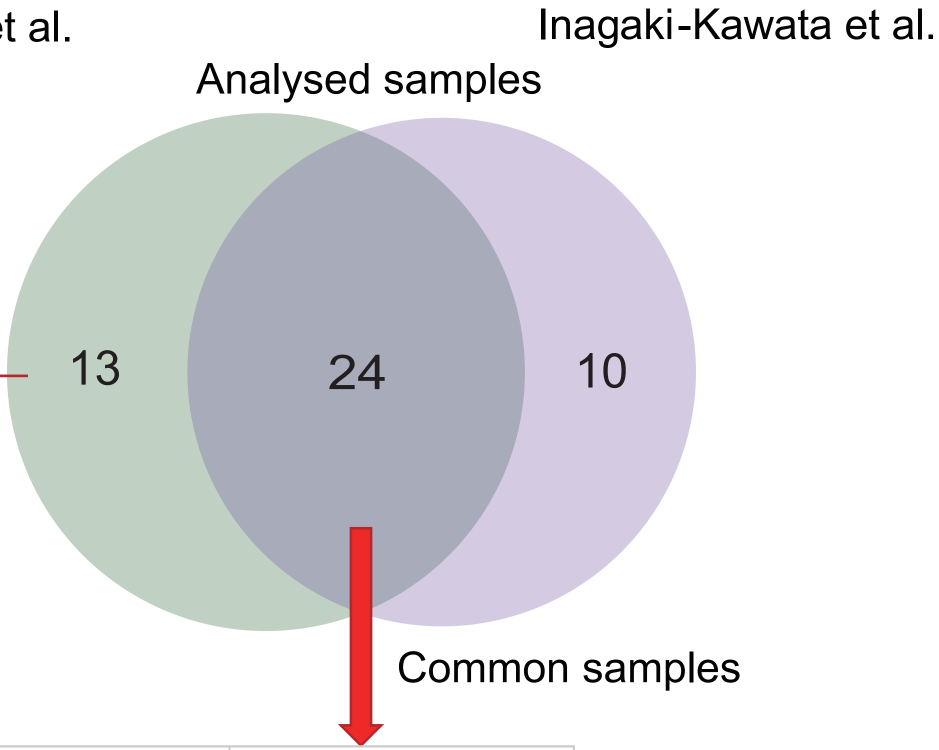

		Inagaki-Kawata et al.	
		Biallelic inactivation(+)	Biallelic inactivation(-)
Maxwell et al.	Biallelic inactivation(+)	20	0
	Biallelic inactivation(-)	2	2

Somatic mutations
TCGA_A1_A0SH
TCGA_A2_A0T0

Supplementary Figure 9

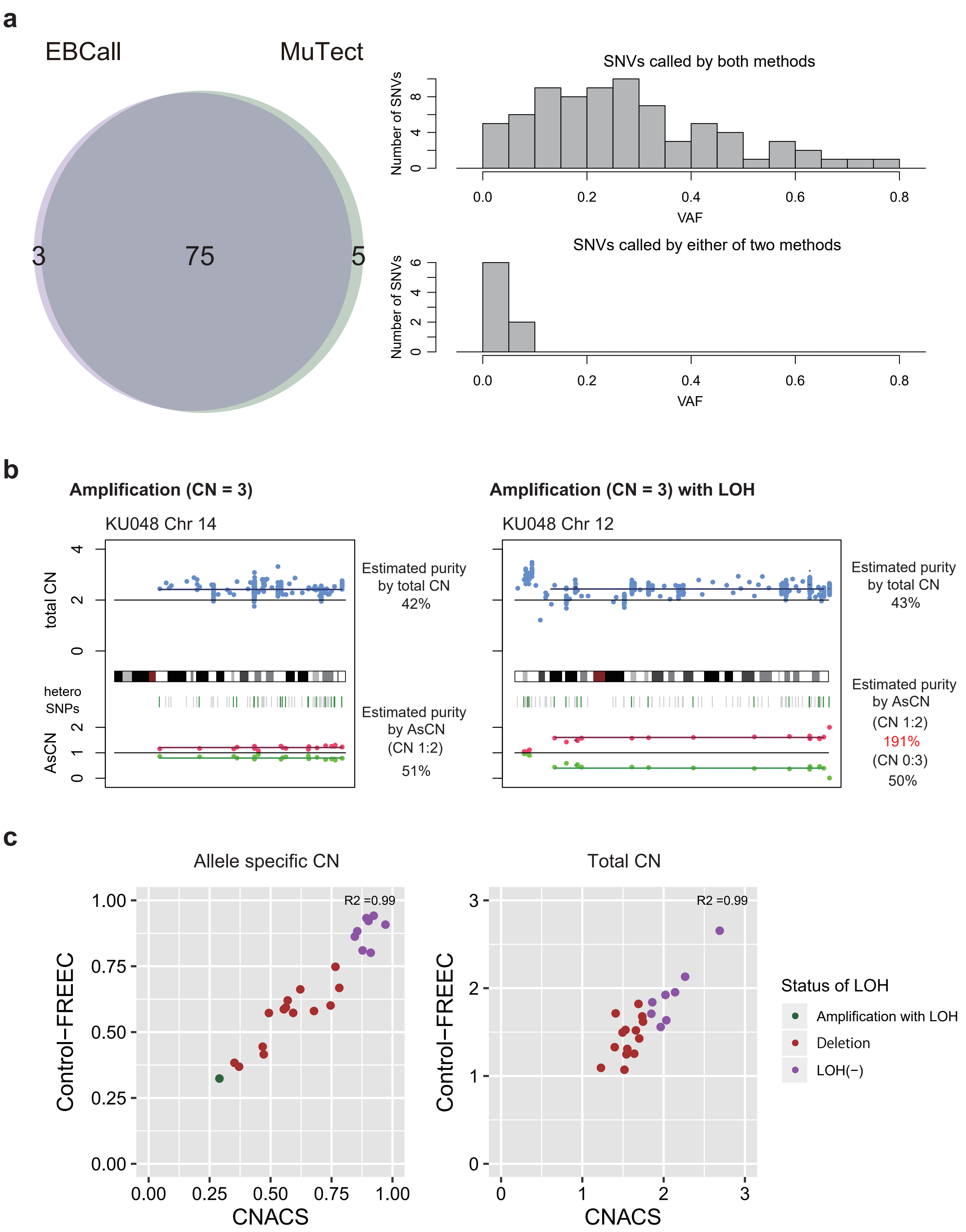

Supplementary Figure 10

KU003

CNACS

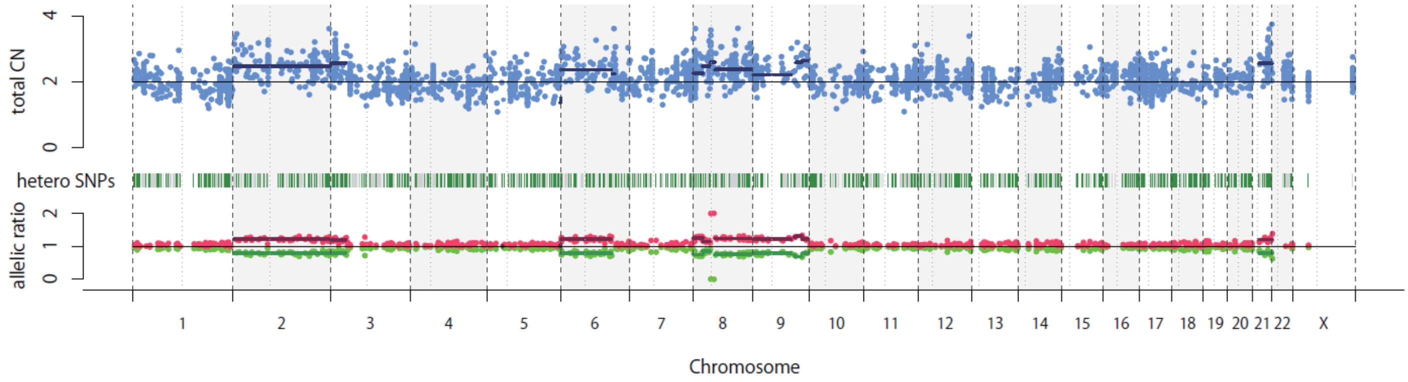

SNP array

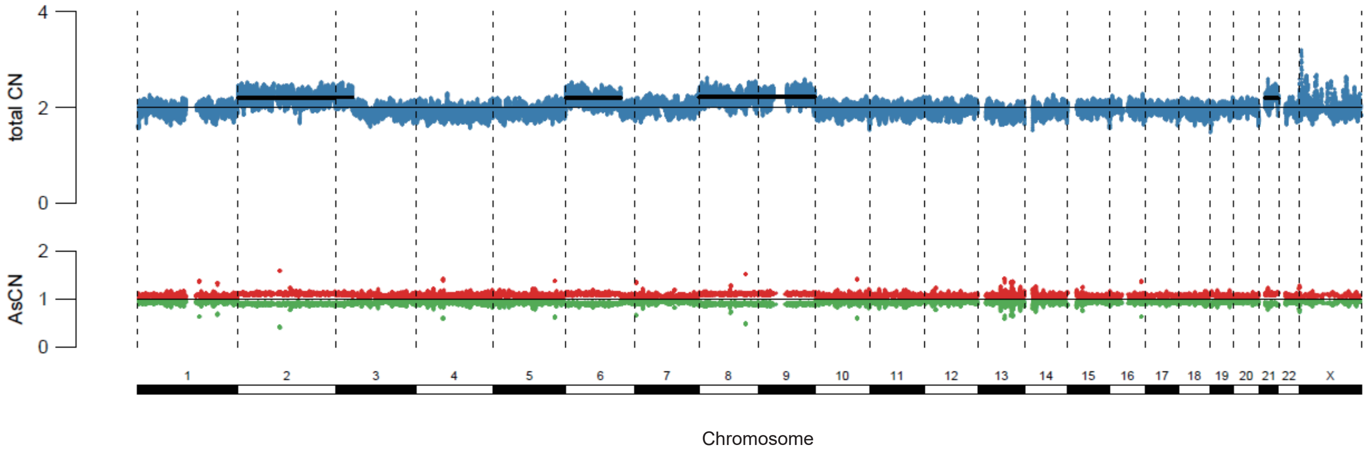

KU006

CNACS

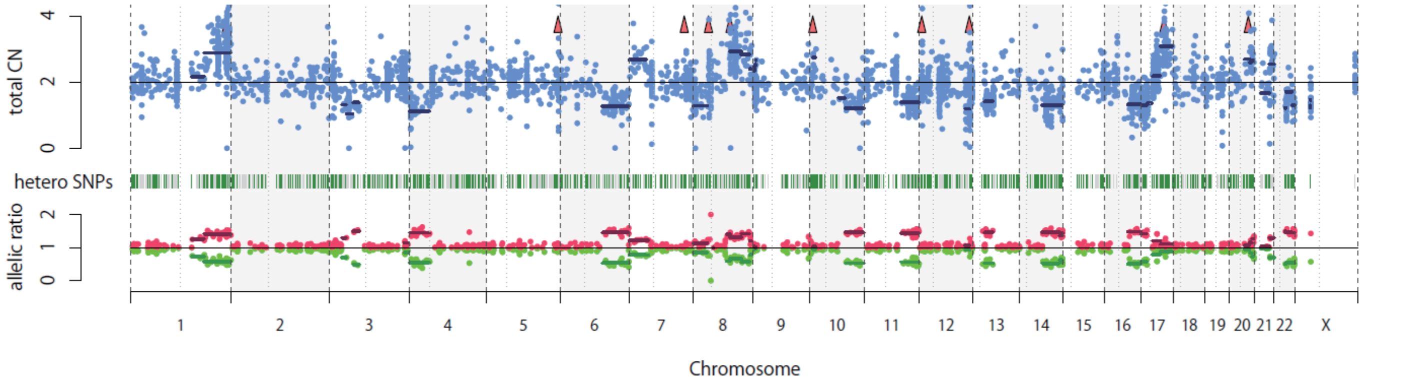

SNP array

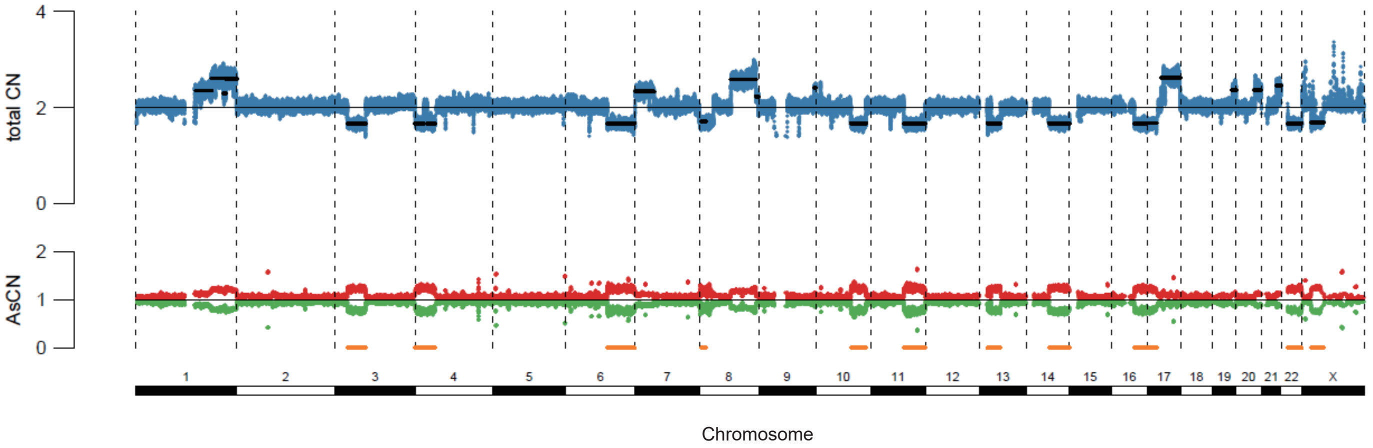

KU012

CNACS

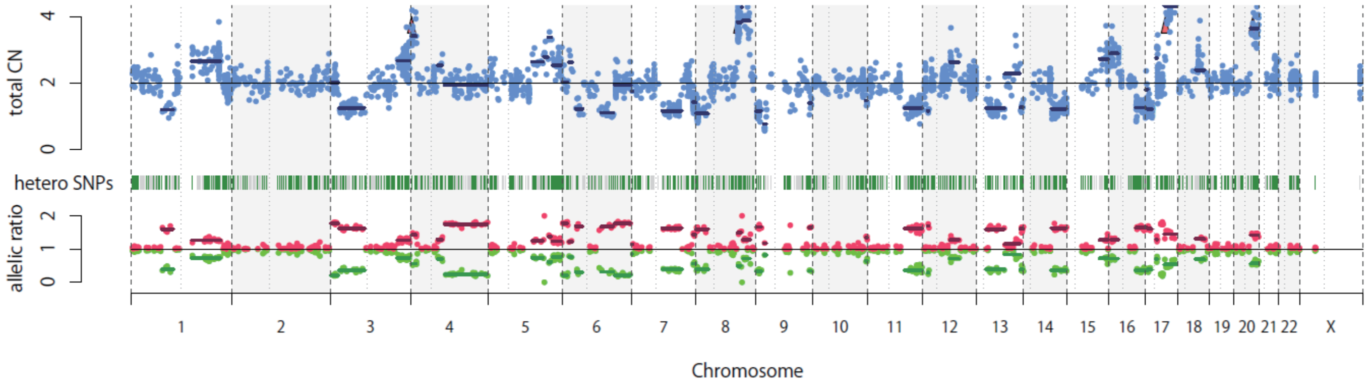

SNP array

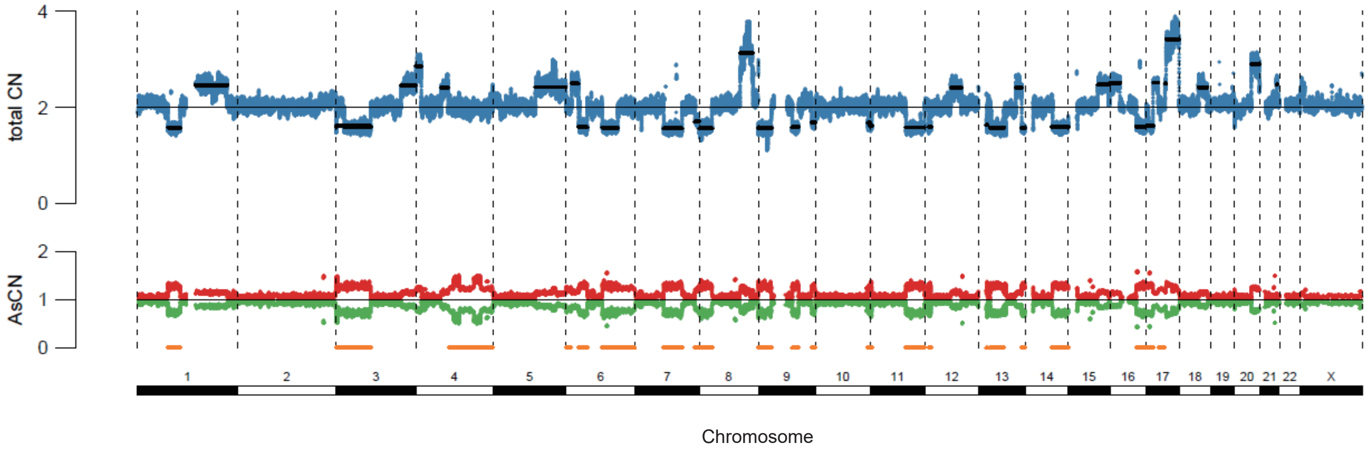

KU014

CNACS

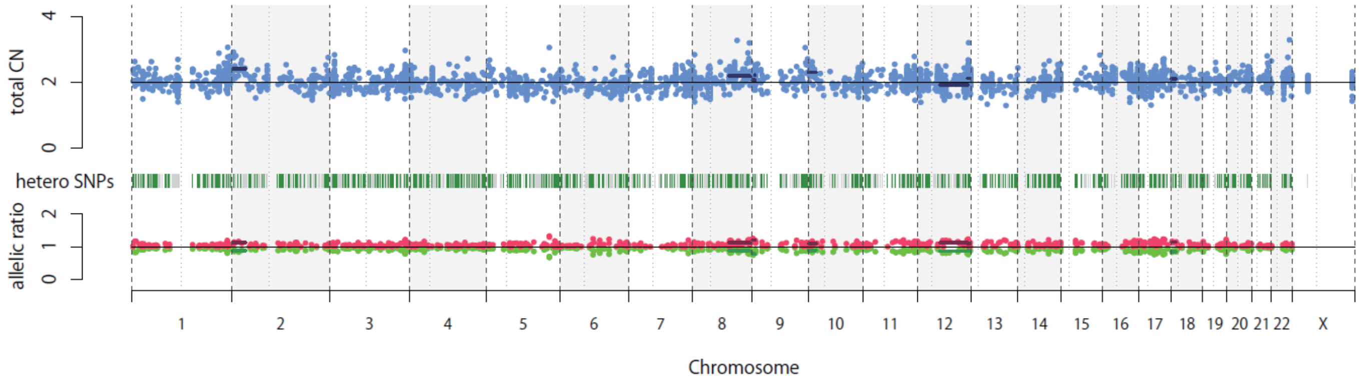

SNP array

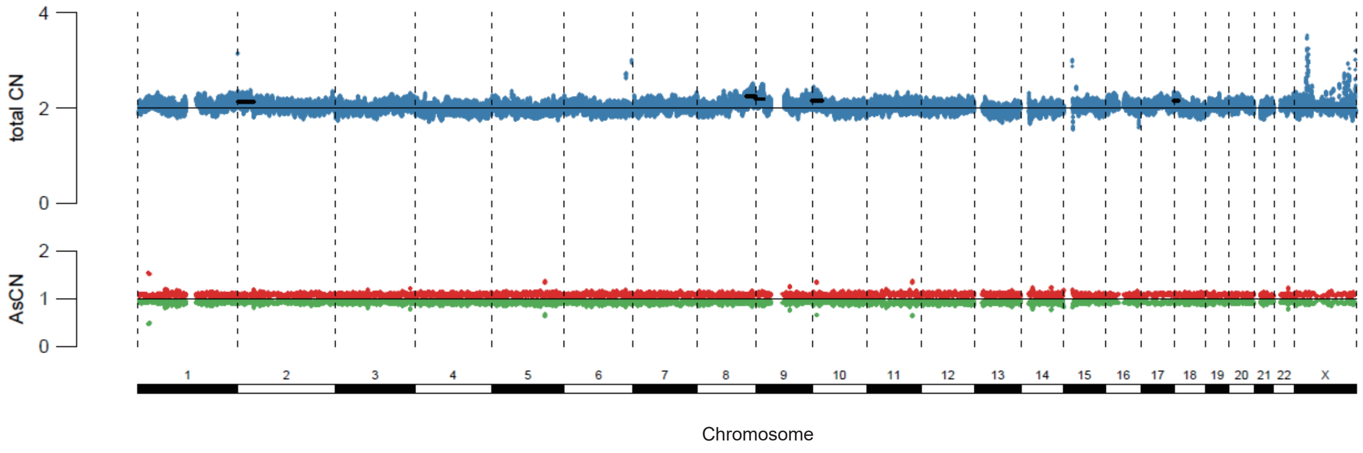

KU028

CNACS

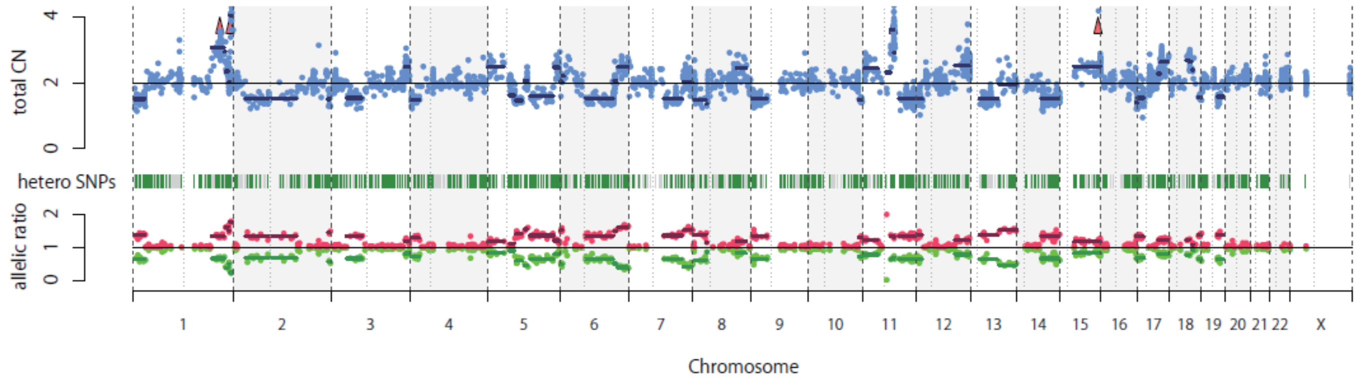

SNP array

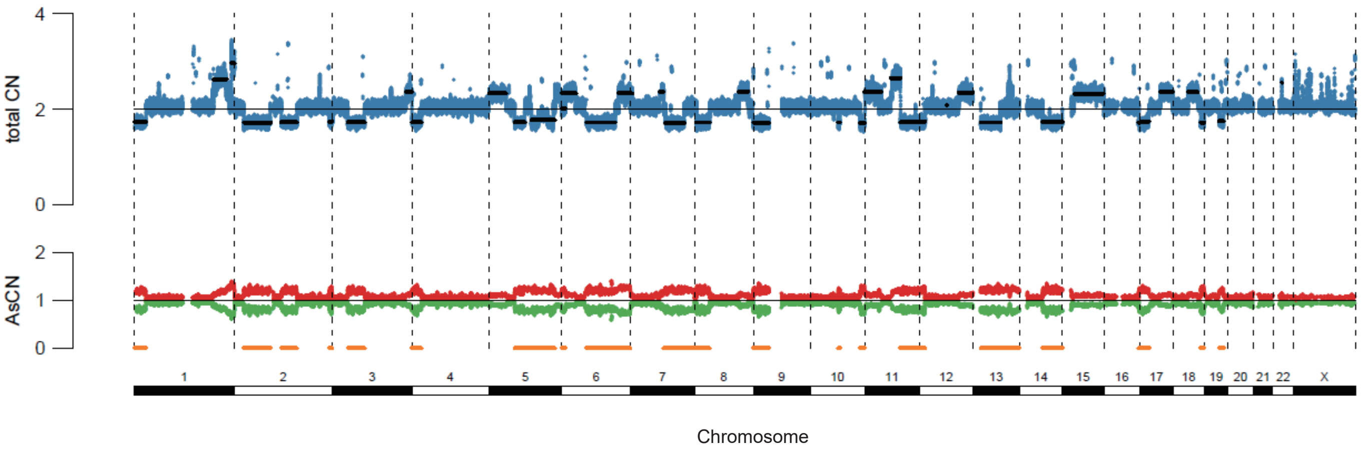

Supplement: Supplementary file 1 — Supplementary Information [file 42003_2020_1301_MOESM1_ESM.pdf]
