# Supplementary material for: Genetic and clinical landscape of breast cancers with germline BRCA1/2 variants
Source: Commun Biol. 2020 Oct 16;3:578. doi: 10.1038/s42003-020-01301-9 (PMC7567851; doi:10.1038/s42003-020-01301-9)
Supplement: Supplementary file 2 — Description of Additional Supplementary Files [file 42003_2020_1301_MOESM2_ESM.pdf]

Description of Additional Supplementary Files

File Name: Supplementary Data 1
Description: Data source of Figure 1b-1d.

File Name: Supplementary Data 2
Description: Data source of Figure 2a-2b.

File Name: Supplementary Data 3
Description: Data source of Figure 3a-3e.

File Name: Supplementary Data 4
Description: Data source of Figure 4a-d.
