# Supplementary material for: Genetic and clinical landscape of breast cancers with germline BRCA1/2 variants
Source: Commun Biol. 2020 Oct 16;3:578. doi: 10.1038/s42003-020-01301-9 (PMC7567851; doi:10.1038/s42003-020-01301-9)
Supplement: Supplementary file 7 — Reporting Summary [file 42003_2020_1301_MOESM7_ESM.pdf]

nature research

Corresponding author(s): Masakazu Toi

Last updated by author(s): Aug 19, 2020

Reporting Summary

Nature Research wishes to improve the reproducibility of the work that we publish. This form provides structure for consistency and transparency in reporting. For further information on Nature Research policies, see our Editorial Policies and the Editorial Policy Checklist.

Statistics

For all statistical analyses, confirm that the following items are present in the figure legend, table legend, main text, or Methods section.

n/a	Confirmed	
☐	☒	The exact sample size (n) for each experimental group/condition, given as a discrete number and unit of measurement
☐	☒	A statement on whether measurements were taken from distinct samples or whether the same sample was measured repeatedly
☐	☒	The statistical test(s) used AND whether they are one- or two-sided<br>*Only common tests should be described solely by name; describe more complex techniques in the Methods section.*
☒	☐	A description of all covariates tested
☒	☐	A description of any assumptions or corrections, such as tests of normality and adjustment for multiple comparisons
☐	☒	A full description of the statistical parameters including central tendency (e.g. means) or other basic estimates (e.g. regression coefficient) AND variation (e.g. standard deviation) or associated estimates of uncertainty (e.g. confidence intervals)
☐	☒	For null hypothesis testing, the test statistic (e.g. F, t, r) with confidence intervals, effect sizes, degrees of freedom and P value noted<br>*Give P values as exact values whenever suitable.*
☒	☐	For Bayesian analysis, information on the choice of priors and Markov chain Monte Carlo settings
☒	☐	For hierarchical and complex designs, identification of the appropriate level for tests and full reporting of outcomes
☒	☐	Estimates of effect sizes (e.g. Cohen's d, Pearson's r), indicating how they were calculated

Our web collection on statistics for biologists contains articles on many of the points above.

Software and code

Policy information about availability of computer code

Data collection	A total of 2,136 breast cancer patients were enrolled in this study who were treated through Kyoto Breast Cancer Research Network institutions, consisting of Kyoto University Hospital and 17 affiliated institutions, from September 2011 to October 2016. Among these, 1,995 cases fulfilled following inclusion criteria: female; sufficient amount of high quality genomic DNA; pathological diagnosis of breast cancer; clinical data of at least one form age of onset, histology, phenotype, grade, clinical stage, past history, and family history. Written informed consent was obtained from all participants. The study was reviewed and approved by the Ethics Committee of Kyoto University Graduate School and Faculty of Medicine and Kyoto University Hospital and was performed in accordance with the Declaration of Helsinki (2013 revision). As for external data sets, we downloaded sequencing data of 829 whole-exome sequencing (WES) of breast cancer tumors from the TCGA Data Portal (https://portal.gdc.cancer.gov/).
Data analysis	Descriptions of the software and analysis have been provided in Online Methods. Sequencing data was processed using our in-house pipeline Genomon2 and MuTect. External bam files were converted to fastq format using biobambam2. Copy number analysis using sequencing data was performed by in-house pipeline CNACS and Control-FREEC. Copy number data using SNP-array karyotyping was performed using CNAG. Mutational signature was evaluated using pmsignature.<br><br>List of programs and softwares:<br>Genomon2 pipeline: version 2.6.0 (https://genomon.readthedocs.io/ja/latest)<br>- Burrows-Wheeler Aligner: version 0.7.10 (https://sourceforge.net/projects/bio-bwa/)<br>- picard-tools: version 1.39 (http://picard.sourceforge.net/)<br>- biobambam2: version 2.0.85 (https://www.sanger.ac.uk/science/tools/biobambam)<br>- GenomonMutationFilter: (https://github.com/Genomon-Project/GenomonMutationFilter)<br>- EBCall: version 2 (https://github.com/friend1ws/EBCall)<br>Integrative Genomics Viewer (IGV): version 2.4.6 (http://software.broadinstitute.org/software/igv/)

CNACS: (https://github.com/papaemmelab/toil_cnacs)
pmsignature: version 0.3.0 (https://github.com/friend1ws/pmsignature)
MuTect: (https://software.broadinstitute.org/cancer/cga/mutect)
Control-FREEC: (http://boevalab.inf.ethz.ch/FREEC/)
CNAG: (http://www.genome.umin.jp/CNAG_DLpage/CNAG_top.html)

For manuscripts utilizing custom algorithms or software that are central to the research but not yet described in published literature, software must be made available to editors and reviewers. We strongly encourage code deposition in a community repository (e.g. GitHub). See the Nature Research guidelines for submitting code & software for further information.

Data

Policy information about availability of data

All manuscripts must include a data availability statement. This statement should provide the following information, where applicable:
- Accession codes, unique identifiers, or web links for publicly available datasets
- A list of figures that have associated raw data
- A description of any restrictions on data availability

Targeted sequencing data of 60 breast cancer samples have been deposited at the European Genome-phenome Archive (https://www.ebi.ac.uk/ega/) under the accession number EGAS00001004182.

Field-specific reporting

Please select the one below that is the best fit for your research. If you are not sure, read the appropriate sections before making your selection.

☒ Life sciences ☐ Behavioural & social sciences ☐ Ecological, evolutionary & environmental sciences

For a reference copy of the document with all sections, see nature.com/documents/nr-reporting-summary-flat.pdf

Life sciences study design

All studies must disclose on these points even when the disclosure is negative.

Sample size	No statistical methods were used to determine sample size since this is an exploratory study. We enrolled as many patients as possible who provided consent for our study during the enrollment period between September 2011 and October 2016. A total of 2,136 breast cancer patients were enrolled in this study. As for external data sets, 829 breast cancers were analyzed.
Data exclusions	We excluded patients with consent withdrawal, DNA samples with low quality/quantity, samples without clinical data and male patients. For the analysis of TCGA samples, samples subjected to whole genome amplification were excluded from analysis to correctly define copy number changes and SVs.
Replication	Sequencing data was processed using Genomon2 and MuTect. Copy number analysis using sequencing data was performed by in-house pipeline CNACS, SNP array karyotyping and Control-FREEC.
Randomization	Not applicable, since this is a case-series study which was therefore not planned to detect any difference in effects between the cohorts with and without intervention.
Blinding	Not applicable, since this is a case-series study.

Reporting for specific materials, systems and methods

We require information from authors about some types of materials, experimental systems and methods used in many studies. Here, indicate whether each material, system or method listed is relevant to your study. If you are not sure if a list item applies to your research, read the appropriate section before selecting a response.

Materials & experimental systems

n/a	Involved in the study	
☒	☐	Antibodies
☒	☐	Eukaryotic cell lines
☒	☐	Palaeontology and archaeology
☒	☐	Animals and other organisms
☒	☐	Human research participants
☐	☒	Clinical data
☒	☐	Dual use research of concern

Methods

n/a	Involved in the study	
☒	☐	ChIP-seq
☒	☐	Flow cytometry
☒	☐	MRI-based neuroimaging

Clinical data

Policy information about clinical studies

All manuscripts should comply with the ICMJE guidelines for publication of clinical research and a completed CONSORT checklist must be included with all submissions.

Clinical trial registration	Not applicable. This study is not a clinical trial.
Study protocol	Not applicable. This study is not a clinical trial.
Data collection	Not applicable. This study is not a clinical trial.
Outcomes	Not applicable. This study is not a clinical trial.
